# Proteogenomic analysis of the autoreactive B cell repertoire in blood and tissues of patients with Sjögren’s syndrome

**DOI:** 10.1136/annrheumdis-2021-221604

**Published:** 2022-02-10

**Authors:** Mathijs G A Broeren, Jing J Wang, Giulia Balzaretti, Patricia J T A Groenen, Barbera D C van Schaik, Tim Chataway, Charlotte Kaffa, Sander Bervoets, Konnie M Hebeda, Gergana Bounova, Ger J M Pruijn, Thomas P Gordon, Niek De Vries, Rogier M Thurlings

**Affiliations:** 1 Department of Rheumatology, Radboudumc, Nijmegen, The Netherlands; 2 Department of Biomolecular Chemistry, Institute for Molecules and Materials, Radboud University, Nijmegen, The Netherlands; 3 Department of Immunology, Flinders University, Adelaide, South Australia, Australia; 4 Department of Clinical Immunology and Rheumatology, Amsterdam Rheumatology and Immunology Center, Amsterdam, The Netherlands; 5 Department of Pathology, Radboudumc, Nijmegen, The Netherlands; 6 Bioinformatics Laboratory, Department of Epidemiology and Data Science, Amsterdam University Medical Centres, Amsterdam, The Netherlands; 7 College of Medicine and Public Health, Flinders University of South Australia, Adelaide, South Australia, Australia; 8 Radboud Technology Center for Bioinformatics, Radboudumc, Nijmegen, The Netherlands; 9 Enpicom BV, ‘s Hertogenbosch, The Netherlands; 10 SA Pathology, Department of Immunology, College of Medicine and Public Health, Flinders University, Bedford Park, South Australia, Australia

**Keywords:** Sjogren's syndrome, B-lymphocytes, autoimmunity, autoantibodies

## Abstract

**Objective:**

To comparatively analyse the aberrant affinity maturation of the antinuclear and rheumatoid factor (RF) B cell repertoires in blood and tissues of patients with Sjögren’s syndrome (SjS) using an integrated omics workflow.

**Methods:**

Peptide sequencing of anti-Ro60, anti-Ro52, anti-La and RF was combined with B cell repertoire analysis at the DNA, RNA and single cell level in blood B cell subsets, affected salivary gland and extranodal marginal zone lymphomas of mucosa-associated lymphoid tissue (MALT) of patients with SjS.

**Results:**

Affected tissues contained anti-Ro60, anti-Ro52, anti-La and RF clones as a small part of a polyclonal infiltrate. Anti-Ro60, anti-La and anti-Ro52 clones outnumbered RF clones. MALT lymphoma tissues contained monoclonal RF expansions. Autoreactive clones were not selected from a restricted repertoire in a circulating B cell subset. The antinuclear antibody (ANA) repertoires displayed similar antigen-dependent and immunoglobulin (Ig) G1-directed affinity maturation. RF clones displayed antigen-dependent, IgM-directed and more B cell receptor integrity-dependent affinity maturation. This coincided with extensive intra-clonal diversification in RF-derived lymphomas. Regeneration of clinical disease manifestations after rituximab coincided with large RF clones, which not necessarily belonged to the lymphoma clone, that displayed continuous affinity maturation and intra-clonal diversification.

**Conclusion:**

The ANA and RF repertoires in patients with SjS display tissue-restricted, antigen-dependent and divergent affinity maturation. Affinity maturation of RF clones deviates further during RF clone derived lymphomagenesis and during regeneration of the autoreactive repertoire after temporary disruption by rituximab. These data give insight into the molecular mechanisms of autoreactive inflammation in SjS, assist MALT lymphoma diagnosis and allow tracking its response to rituximab.

**Key messages**

**What is already known about this subject?**

- Sjögren’s syndrome features activated B cells in affected tissues, aberrancies in circulating B cell populations and autoantibodies, including anti-Ro60/SSA, antiRo52/SSA, anti-La/SSB and rheumatoid factors (RFs).

**What does this study add?**

- RF and antinuclear antibody (ANA) clones are enriched in affected tissues where they occur as a small part of a polyclonal repertoire. RF and ANA clones affinity maturate in divergent fashion, which increases during secondary RF lymphomagenesis and after temporary disruption by rituximab (RTX).

**How might this impact on clinical practice or future developments?**

- Analysis of RF clones in affected tissues may assist identification of mucosa-associated lymphoid tissue lymphomas and tracking of their response to RTX.

## Introduction

Sjögren’s syndrome (SjS) is a systemic autoimmune disease, principally affecting exocrine glands. It features activated B cells in affected tissues, aberrancies in circulating B cell populations and circulating autoantibodies (AutoAbs), including antinuclear antibodies (ANAs) anti-Ro60/SSA, anti-Ro52/SSA, anti-La/SSB and rheumatoid factors (RFs).^1–3^ Although the precise role of autoreactive B cells in the pathogenesis of SjS is less well defined, the pathogenic role of these AutoAbs is suggested by animal experiments^4 5^ and clinical observations.^6^ Antibodies produced by lymphomas that develop in up to 10% of patients with SjS, most commonly mucosa-associated lymphoid tissue (MALT) lymphomas, express immunoglobulins (Igs) with RF activity.^7–10^

The generation of the ANA-specific and RF-specific B cell repertoires and how they breach self-tolerance checkpoints has not been precisely determined. In experimental animal models, autoreactive B cells affinity maturate in antigen-dependent fashion in germinal centres in lymphoid tissues or in extrafollicular sites such as the splenic marginal zone.^11–13^ In some models, this involves stochastic selection of follicular B cells after an environmental stimulus in a genetically predisposed host. In other models, autoreactive B cells are generated from extrafollicular, polyreactive precursor B cells.^14^ The generation of antinuclear antigen-reactive B cells in affected tissues of patients with SjS has been related to positive selection of polyreactive precursor B cells.^15 16^ Selection of these clones was enhanced by N-glycosylation sites in the B cell receptor variable region, resulting in activation by C-type lectins.^16^ RF clones have been suggested to be selected from extrafollicular precursors.^17^

Evidence suggests that the same mechanisms operative in SjS might also contribute to generation of SjS-associated RF-derived MALT lymphomas. Lymphomagenesis of RF clones results from gradual accumulation of lymphoma driver mutations.^10^ These lymphomas are clonally related to reactive B cell aggregates in the same salivary gland (SG). The latter are frequently organised as ectopic germinal center (GC)-like structures and display functional features.^18 19^ High levels of somatic hypermutation (SHM) and intra-clonal variation in these lymphomas suggest that ectopic GCs allow RF B cell clones to proliferate and maturate, resulting in somatic mutations and MALT lymphoma development.^18 20–22^ It is unknown why the RF repertoire is prone to oncogenic transformation compared with the ANA repertoire. MALT lymphomas can respond well to rituximab (RTX) mediated B cell depletion, but will eventually relapse.^8^ Hypothetically, the ANA and RF repertoire regenerates in a different manner together with the lymphoma clones.

Herein, we combined mass spectrometry (MS) approach for serum antibody sequencing with methods to analyse the B cell repertoire at the RNA, DNA and single cell level to investigate the selection and affinity maturation of the autoreactive B cell repertoire in blood and tissues of patients with SjS.

## Methods

### Study subjects

The autoreactive B cell repertoire was analysed in six patients with SjS, fulfilling the 2016 ACR-EULAR classification criteria, in comparison to 4 age-matched and 3 elderly healthy controls (table 1). Among patients with SjS, B005 and B007 developed MALT lymphoma and were treated with RTX. An overview of the blood and tissue sampling for all six patients is shown in online supplemental figure 1. Two additional patients, B012 and B013, were followed by B cell clonality testing of biopsies before and during diagnostic work up for MALT lymphoma diagnosis. The B cell repertoire in blood was analysed in 24 patients with SjS, fulfilling the 2016 ACR-EULAR classification criteria, 10 disease controls (systemic lupus erythematosus (LE), subacute cutaneous LE, systemic sclerosis and rheumatoid arthritis) that tested positive for anti-Ro and/or anti-La antibodies and 24 healthy controls (table 2). All study subjects gave written informed consent prior to inclusion in the study. For more details about the study subjects, see online supplemental methods.

10.1136/annrheumdis-2021-221604.supp1 Supplementary data

10.1136/annrheumdis-2021-221604.supp12 Supplementary data

**Table 1:** Patient characteristics at time of first study biopsy

Patient code	Sex	Age (years)	Duration since diagnosis (years)	IgM-RF titre (ie, /mL, cut-off: 10 mL)	IgG titre (g/L, cut-off: 16 g/L)	ESSDAI
B005	F	59	30	170	18	4
B007	M	63	7	4500	25	11
B008	M	61	1	48	19	6
B009	F	30	10	280	26	2
B010	F	74	21	15	15	2
B011	F	46	2	148	22	3
HC1	F	59	–	–	–	–
HC2	F	60	–	–	–	–
HC3	F	23	–	–	–	–
HC4	M	30	–	–	–	–
HC5	M	81	–	–	–	–
HC6	F	82	–	–	–	–
HC7	F	80	–	–	–	–

ESSDAI, EULAR Sjögren’s Syndrome Disease Activity Index.

**Table 2:** Clinical and disease characteristics and B cell repertoire features in patient groups

	SjS (n=26)	Disease controls (n=10)	Healthy controls (n=24)
Female, %	73	70	46
Age in years, mean±SD	54.4±15.3	57±11.8	56.6±14.0
Diagnosis disease controls			
SLE, n	–	5	–
SCLE, n	–	3	–
Other, n	–	2	–
Individuals tested for virology, n	24	6	21
CMV-IgG positive, %	42	50	38
VCA-IgG positive, %	92	100	86
EBNA-IgG positive, %	79	67	76
Anti-SSB, n	22	8	–
Anti-SSA, n	26	9	–
RF, n	16	2	–
RF titres in IU/mL, median (IQR)	100 (41.3–207.5)	101 (11–191)	–
SLE antibodies, n	5	4	–
Anti-CCP, n	2	1	–
Individuals tested for serum IgG, n	23	3	–
IgG positive, %	60.9	66.7	–
IgG titres in IU/mL, mean±SD	20.9±4.8	19±7.8	–
Serum cryoglobulins, n	3	1	–
Serum M protein, n	1	0	–
Leucopenia, n	9	2	–
Neutropenia, n	3	1	–
Lymphopenia, n	7	4	–
ESSDAI, median (IQR)	2 (1–2.3)	1 (0–2)	–
Maximal past ESSDAI, median (IQR)	3 (2–5.3)	1.5 (0.3–8)	–
ESR in mm/hour, median (IQR)	20 (7.5–28.5)	8 (2–43)	
Concomitant Raynaud, n	15	5	–
Concomitant arthritis, n	4	3	–
Organ involvement, n	5	3	–
WB HES, median (IQR)	2.5 (0.8–6.5)	3.5 (0–7.8)	0.5 (0–4.8)
Percentage of clones with SHM >0 in WB, median (IQR)	60.5 (50.8–75.6)	69.2 (57.6–81.3)	60.1 (52.7–65)
Percentage of clones with N-glycosylation sites >0 in WB, median (IQR)	6 (5–7.6)	7.8 (5.7–9.5)	5.6 (5.2–6.5)
CDR3 length in WB, mean±SD	24±0.4	23.8±0.4	24.3±0.1

CCP, cyclic citrullinated peptides; CDR3, complementarity determining region 3; CMV, cytomegalovirus; EBNA, EBV nuclear antigen; ESR, erythrocyte sedimentation rate; ESSDAI, EULAR Sjögren's Syndrome Disease Activity Index; HES, highly expressed sequences; Ig, immunoglobulin; RF, rheumatoid factor; SCLE, subacute lupus erythematosus; SHM, somatic hypermutation; SjS, Sjögren’s syndrome; SLE, systemic lupus erythematosus; VCA, virus capsid antigen; WB, whole blood.

### Overview of analysis of Igs in blood and tissues

In six patients and four age-matched and three elderly healthy controls, the B cell repertoire was determined by heavy chain Ig mRNA analysis using next-generation sequencing (NGS; Ig-RNAseq) in blood and tissue samples. In blood samples, a comparison was made between whole blood samples and sorted B cell subsets. This was done to account for the bias toward abundant plasmablast/-cell reads in the blood samples. Sorting of B cells from tissues for clonality is challenging and cannot be performed on stored tissue samples. Therefore, in affected patient tissues, we made a comparison between Ig-RNAseq and NGS at the DNA level for Ig heavy and light chain (Ig-DNAseq) that we validated for clonality testing.^23^ This protocol allows to determine the presence of clonal expansions, B cells with non-productive rearrangements and compensates for the mRNA abundance in plasma cells compared with memory B cells. We analysed two tissues at clinical relapse after RTX using a combination of bulk Ig-RNAseq and 10× Genomics general and Ig single cell RNA sequencing (sc-RNAseq and Ig-sc-RNAseq). Finally, we analysed the prevalence of autoreactive sequences in the overall B cell repertoire using MS sequence analysis (MS-seq). For this, we affinity-purified four generally prevalent AutoAbs in SjS from serum and analysed their complementarity determining region 3 (CDR3) amino acid sequences. The resulting MS sequences for anti-Ro60, anti-Ro52, anti-La and RF were aligned after blinding with all heavy chain Ig-RNAseq, Ig-DNA-seq and Ig-sc-RNAseq data from blood and tissue samples for the mapping of autoreactive peptide sequences of the same patient. The antigen-specific B cell clones in the circulation and tissues were then identified based on the matched CDR3 peptides. An overview of the methods performed for each sample is provided in online supplemental figure 1. Online supplemental figure 2 presents an overview of analyses performed in patients B005 and B007, the two patients with MALT lymphoma that were treated with RTX and followed in time. For more details about the experimental and bioinformatic methods, see online supplemental methods.

10.1136/annrheumdis-2021-221604.supp2 Supplementary data

## Results

### Memory B cell subsets in blood and tissues of patients with SjS contain expanded clones

**Figure 1:**
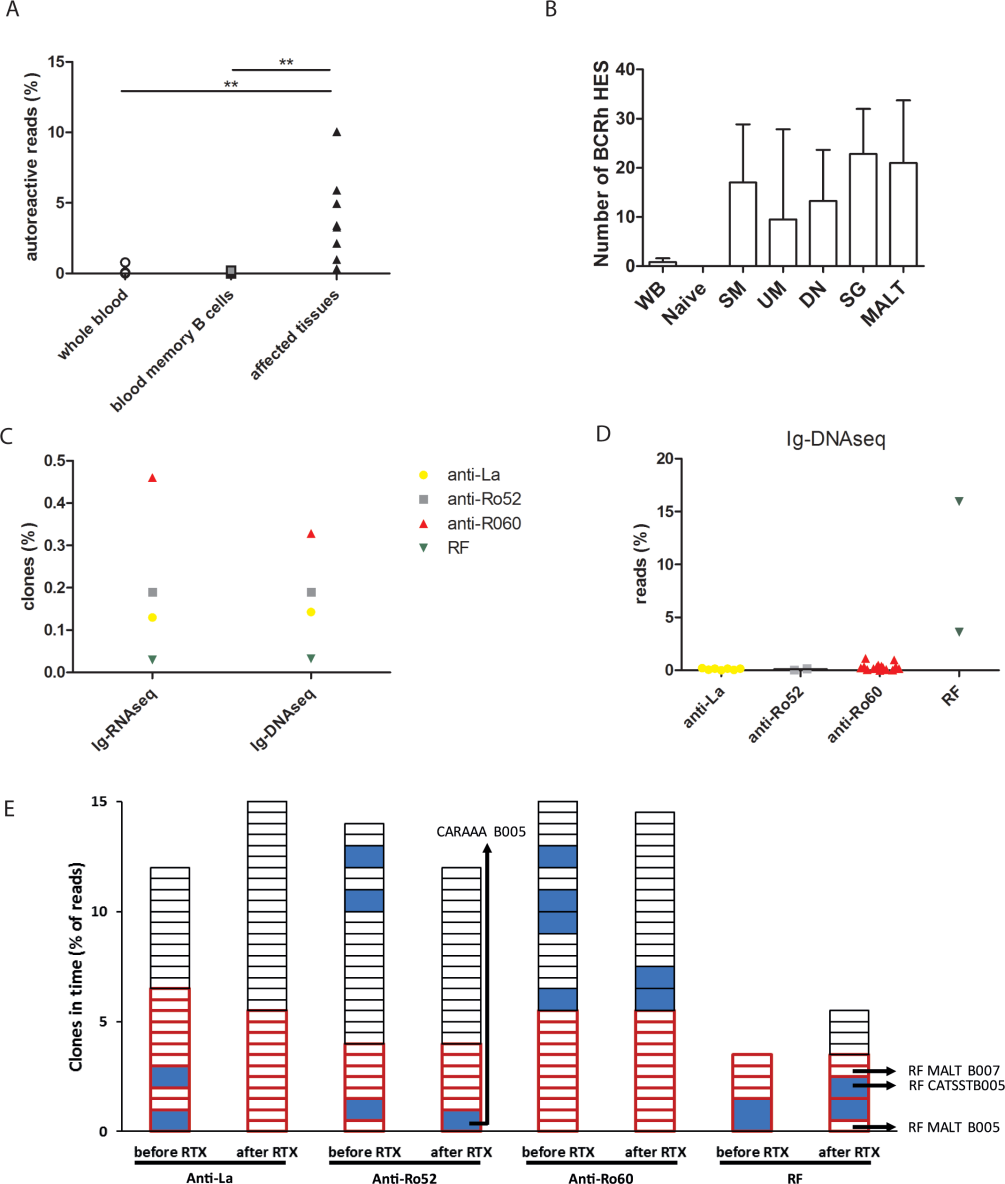
Analysis of autoreactive B cell clones in patients with SjS. (A) The presence of autoreactive B cell clones in blood versus tissues of six patients with SjS. The percentage of autoreactive Ig-RNA reads of all Ig from the same compartment per patient is shown. WB and sorted memory B cell subsets from blood were compared with affected tissues. Pooled analyses are shown for the sorted UM, DN and SM B cell subsets and for collected tissues (SG and MALT lymphoma); (B) the presence of HESs in WB, naive and memory B cell subsets sorted from blood and SG and MALT tissues. Sorted cells were UM B cells (CD19 +, CD38−, IgD+ and CD27+), DN B cells (CD19 +, CD38−, IgD− and CD27−) and SM B cells (CD19 +, CD38−, IgD− and CD27+); (C) mean percentage of clones in affected tissues that mapped to proteomic sequences of serum anti-Ro52, anti-La, anti-Ro60 and RF AutoAb per patient comparing Ig-RNAseq and Ig-DNAseq; (D) Ig-DNAseq analysis of the percentage of autoreactive reads of all Igs from the same tissue in MALT lymphoma tissues; (E) the presence of autoreactive clones in SG and MALT tissues before and after RTX for patients B005 and B007 in Ig-RNAseq. Small squares indicate clones of up to 0.5% of reads. Larger squares (in blue) depict larger clones with number of reads rounded off to multiples of 0.5%. The legend depicts the first amino acids of the CDR3 sequence of large clones of interest. Squares with red bold border line indicate clones that are detected before and after RTX. Clones were defined by sequences with the same V and J segments and the same CDR3 region. Yellow dots show anti-La, grey squares anti-Ro52, red triangles anti-Ro60, green squares RF and dark-green MALT. Panels A shows medians and IQRs. **p<0.01. AutoAb, autoantibodies; CDR3, complementarity determining region 3; DN, double-negative; HESs, highly expressed sequences; Ig, immunoglobulin; MALT, mucosa-associated lymphoid tissue; RF, rheumatoid factor; RTX, rituximab; SG, salivary gland; SjS, Sjögren’s syndrome; SM, switched memory; UM, unswitched memory; WB, whole blood.

First, we analysed the anti-Ro/anti-La and RF repertoire in blood and affected tissues from six patients with SjS. Ig-RNAseq showed that a higher fraction of Ig reads from affected tissues compared with blood mapped to MS proteomic sequences of AutoAbs (p<0.01; figure 1A).

To gain more insight in the B cell repertoire in blood of patients with SjS with anti-Ro/anti-La, we compared these (n=26) with patients with other anti-Ro/anti-La positive autoimmune disease (n=10) and healthy controls (n=24). Most Ig sequences detected in blood were low abundant clones, but a number of sequences was highly expressed (HES: >0.5% of total Ig reads, based on previous observations^24^) (figure 1B; online supplemental table 1 for total reads). The number of HESs did not differ significantly between the groups or in patients with RF, although there was a numerical trend toward a higher number of HESs in disease groups compared with healthy controls (table 2). Other repertoire features, that is, the extent of SHM, CDR3 characteristics and the number of potential N-glycosylation sites, were also similar between groups.

We compared the repertoire in blood and affected tissues in the six patients with SjS in whom biopsies were acquired. HESs detected by Ig-RNAseq are predominantly expanded plasmablasts/-cell clones, since these produce large amounts of Ig compared with memory B cells. Therefore, we compared HESs in whole blood samples to sorted memory B cell subsets. HESs were detected in unswitched memory (UM), switched memory (SM) and double-negative (DN) B cells in contrast to naïve B cells (figure 1B).

Tissues contained a similar number of HESs compared with circulating memory B cell subsets. The SHM load was similar between circulating memory B cell subsets and affected tissues (online supplemental figure 4A). The number and SHM load of HESs were lower in whole blood in line with a larger proportion of naïve B cells in those samples. The overlap between the clones in the tissues and in the circulating subsets was low, both for the complete set of Ig sequences and for the HESs (online supplemental figure 4B, C). The overlap in HESs was highest between tissue and whole blood, possibly because of circulating plasma cells in the latter samples. In only one patient, there was 30% overlap between HESs in SG tissue and circulating SM B cells. In summary, circulating memory B cell subsets contain expanded clones, but these only occasionally concern clones from affected tissues.

10.1136/annrheumdis-2021-221604.supp4 Supplementary data

### Tissues are enriched for autoreactive clones

We analysed the relationship between HESs and AutoAbs in blood. A small proportion of the B cell repertoire in whole blood and sorted B cell subsets of patients with SjS mapped to amino acid sequences of AutoAbs (figure 1A). RFs were present in the UM and DN populations and ANAs in SM and DN populations. None of the HESs in whole blood or sorted B cell subsets mapped to AutoAbs. AutoAb titres were not correlated to the number of HESs or AutoAb reads. Taken together, memory B cell subsets in blood of patients with SjS contain expanded clones, but these do not concern anti-Ro52, anti-Ro60, anti-La or RF clones.

### Affected tissues contain a polyclonal repertoire, including ANA and RF clones and monoclonal RF expansions in MALT lymphoma tissues

To gain more insight in the autoreactive repertoire in affected tissues, we comparatively analysed clones with immunohistochemistry (IHC), Ig-RNAseq and Ig-DNAseq using a protocol that we developed and validated for detection of B cell clonality by NGS in stored tissues.^23^ HESs in Ig-DNAseq are equally likely to be memory B cell or plasma cell clones. IHC with B cell and plasma cell markers aids differentiation between B cell and plasma cell expansions.

Morphology/IHC for CD20, CD79a, kappa and lambda showed a variable infiltration by B cell follicles (CD20+) and plasma cells (CD79a+/CD20−) in affected SG tissues (online supplemental figure 3). The MALT lymphoma tissues showed more than 50% CD20+ B cells and variable plasma cell infiltration.

10.1136/annrheumdis-2021-221604.supp3 Supplementary data

Both Ig-RNAseq and Ig-DNAseq showed the presence of a polyclonal repertoire with a variable extent of HESs in all tissues. ANA and RF clones constituted a small fraction of clones (figure 1C). MALT lymphoma tissues of B005 and B007 showed a highly expanded B cell clone in Ig-DNAseq based on the presence of clonal Ig heavy and light chain rearrangements. The most abundant Immunoglobulin Heavy Locus framework 3 (IGH-FR3) clonotype in both MALT lymphoma tissues was detected at 3% and 11% of all IGH clonotypes (B005 and B007, respectively (figure 1D; online supplemental data)), which is less than the estimated tumour load. This was probably caused by impaired primer annealing because of SHM of the IGHV–IGHJ rearrangements. In both MALT lymphomas, clonal light chain rearrangements were detected in higher abundance than the IGH-FR3 clonal rearrangement (B005: 64% and B007: 28% (online supplemental data)), which can be explained by the absence of SHM in the Immunoglobulin kappa (IGK) locus: clonal IGKV–IGKJ rearrangements (in B007) do hardly and an intron K-deleting element rearrangements (in B005) do not undergo SHM. The expanded clones mapped to serum MS-seq RF in both patients. In summary, ANA and RF clones are enriched in affected tissues as a small fraction of a polyclonal infiltrate. The analysed MALT lymphomas were monoclonal RF expansions.

### At relapse after RTX treatment, RF clones can occur as a mix of small and large clones

We analysed the regeneration of the autoreactive repertoire in affected tissues after temporary perturbation with RTX monotherapy in two patients with MALT lymphoma (see online supplemental methods). Ig-RNAseq analysis at relapse after RTX showed that the RF lymphoma clone in B005 was only detectable as a small clone (figure 1E). Seven other RF clones occurred, two of which were large. Four of the RF clones were new, three were already detectable before RTX (figure 1E). In B007, the single RF lymphoma clone persisted as a single RF clone.

Similarly, the ANA clones persisted or disappeared after RTX and new clones appeared (figure 1E). Similar to affected tissues before RTX, most ANA clones were small clones and some large, occurring as a small proportion of a polyclonal infiltrate.

### RF and ANA repertoires show stochastic selection

**Figure 2:**
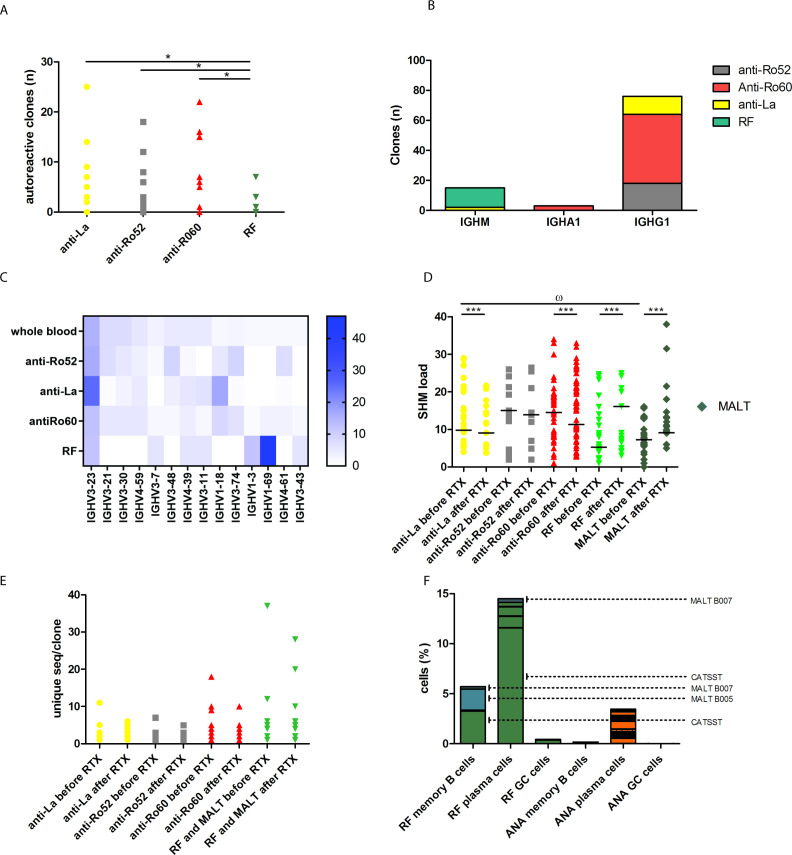
Analysis of selection and affinity maturation of autoreactive B cell clones. Yellow dots show anti-La, grey squares anti-Ro52, red triangles anti-Ro60, green squares RF and dark-green MALT. (A) The number of clones that mapped to sequences of serum anti-Ro52, anti-La, anti-Ro60 and RF AutoAb in all tissue samples before and after RTX. *p<0.05.; (B) isotype of the autoreactive clones of which the constant domain could be successfully attributed for pooled samples; (C) heatmap of all IGHV genes of whole blood samples versus anti-Ro52, anti-Ro60, anti-La and RF clones obtained from pooled samples; (D) SHM load of autoreactive clones before versus after RTX in pooled samples in Ig-RNAseq. Bars show medians: ^ω^p<0.05 for post-hoc Bonferroni-corrected comparisons between all groups before RTX. Medians are shown as: ***p<0.001. (E) Intra-clonal variation of autoreactive clones before versus after RTX in pooled samples in Ig-RNAseq. Unique sequences per clonotype are defined by sequences with the same V and J segments and the same CDR3 region, allowing for two mismatches in CDR3 in Ig-RNAseq. (F) sc-RNAseq was performed in duplo in unselected cells retrieved from biopsies of tissues affected by MALT lymphoma in patients B005 (SG) and B007 (lymph node). Samples were obtained at disease relapse after RTX. T-distributed stochastic neighbor embedding (t-SNE) mapping was performed that identified memory B cells (CD79A+ and CD138−), plasma cells (CD79A+ and CD138+) and germinal center (GC)-like B cell (CD79A+ and MKI67+) clusters. Depicted are the number of cells for each autoreactive clone in these clusters of the pooled samples. Green panels show data for RF clones, blue for MALT RF clones and orange for ANA clones. The largest RF clone in the sample of B005 is shown in the legend with the first amino acids of its CDR3 (CATSST). ANA, antinuclear antibody; AutoAb, autoantibodies; CDR3, complementarity determining region 3; Ig, immunoglobulin; MALT, mucosa-associated lymphoid tissue; RF, rheumatoid factor; RTX, rituximab; sc-RNAseq, single cell RNA sequencing; SG, salivary gland; SHM, somatic hypermutation.

We next analysed gene segment usage of ANA and RF clones in pooled samples. The number of anti-Ro60, anti-La and anti-Ro52 clones was higher than the number of RF clones (figure 2A; online supplemental figure 4D)(p<0.05). All ANA clones of which the isotype could be determined used an IgG1 constant domain, with the exception of two anti-La IgM and two anti-Ro60 IgA1 clones (figure 2B). In contrast, all RF antibodies were IgM.

Analysis of IGHV gene use revealed that RF clones used a limited number of IGHV gene segments with predominant use of IGHV1-69, combined with IGHJ4 and an IGKV3-20 light chain (figure 2C). IGHV1-69, IGHJ4 and IGKV3-20 were used by the MALT lymphomas and the large RF clones that expanded after RTX. This is a stereotypic RF clonotype for SjS-associated MALT lymphoma.^25^ In mice, RFs were generated from extrafollicular B cells from the splenic marginal zone that circulate as UM B cells.^13 26^ In our cohort, expanded clones in UM B cells were not shared with other memory B cell subsets, indicating a unique origin (online supplemental figure 5B). However, shared stereotypic RF sequences were not enriched in UM B cells or their HESs in blood of healthy controls or the same patients with SjS (online supplemental figures 7 and 8). This implies that stereotypic RF selection is driven stochastically by tissue-specific factors.

10.1136/annrheumdis-2021-221604.supp5 Supplementary data

10.1136/annrheumdis-2021-221604.supp7 Supplementary data

10.1136/annrheumdis-2021-221604.supp8 Supplementary data

Earlier studies suggest that ANA may be derived from polyreactive B cell precursors that share sequence motifs.^14–16^ In line with a polyreactive nature, 13% of anti-Ro60, anti-Ro52 and anti-La clones precipitated with 2 out of 3 ANA (online supplemental table 2). ANA clones shared a preference for IGHV3-23, IGHV1-18, IGHV3-74 and IGHV4-61 usage in their variable domains, in line with our previous studies.^27 28^ This preference diverged from RF and the IGHV usage in whole blood samples (figure 2C). The IGHV segments preferred by ANA were not enriched in naïve or memory B cell subsets or expanded clones in blood of patients with SjS vs healthy controls (online supplemental figures 4–6). Taken together, ANA display signs of polyreactivity and similar stochastic tissue-restricted selection that differs from RF clones, despite recognising different antigens.

10.1136/annrheumdis-2021-221604.supp6 Supplementary data

### RF MALT lymphoma clones and large non-MALT RF clones at relapse after RTX show continuing SHM and high intra-clonal diversification

Analysis of affinity maturation showed that the SHM load of RF clones was relatively low compared with ANA clones (figure 2D). The SHM load was somewhat higher in MALT RF compared with other RF clones. After RTX, large RF and MALT RF clones accumulated additional SHM, while the SHM load of ANA clones decreased (figure 2D). This indicates that SHM continues in MALT lymphoma clones and large non-MALT RF clones after RTX.

**Figure 3:**
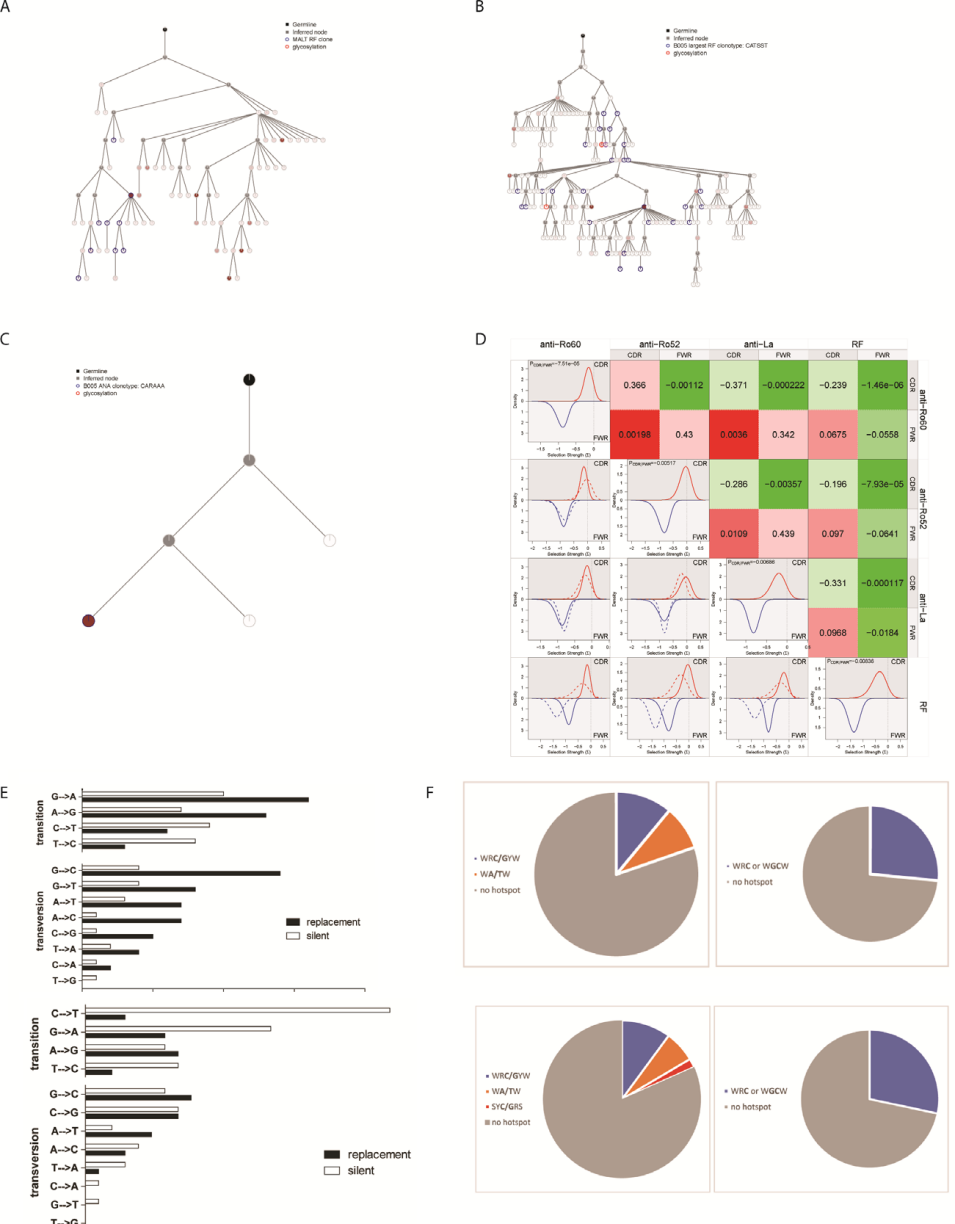
Mutation patterns of affinity maturation of autoreactive B cell clones. (A–C) Lineage tree analysis of the B007 MALT RF clonotype (CAREMD, 86 cells (A)), the B005 largest RF clonotype (CATSST, 519 cells (B)) and the B005 single large ANA clonotype (CARAAA anti-Ro52, 79 cells (C)) in the sc-Ig-RNAseq of patients B005 and B007 tissues at clinical relapse after RTX. Clonotypes are defined as clones with a similar V(D)J assignment to the heavy and light chains and a maximum of two different amino acid mutations. The upper node (germline) depicts the putative germline. The analysis is based on complete Ig sequences. Grey nodes (inferred node) are inferred sequences not observed in the dataset. Blue circles (clonotype node) are all nodes which are assigned to the same clonotype. The colour gradient of the inside of clonotype nodes is a gradient that reflects the number of sequences observed which this exact receptor sequence. The lightest colour (white) in the range represents 1 sequence and the darkest colour represents the maximum number of sequences for the clone (13 sequences for (A), 74 sequences for (B) and 163 sequences for (C)). In addition, Ig sequences with predicted glycosylation sites are shown in a red circle. (D) BASEline analysis of the selection strength of anti-Ro60, anti-Ro52, anti-La and RF clones. All fully sequenced autoreactive clones from B005 and B007 were included in the analysis. The CDR3 sequences were excluded. The lower left graphs depict comparisons of the selection strengths of the CDR and FWR. Each panel shows a comparison between two AutoAbs groups. A low selection strength can result from a high quantity of silent mutations compared with amino acid-changing mutations. The values in coloured fields on the upper right indicate p values for the comparisons in selection strength on the CDR and FWR between AutoAb groups. (E) Total quantities of silent and replacement transitions and transversions in the largest CATSST RF clonotype Ig sequences of B005 (top) and the large MALT CAREMD RF clone of B007 (bottom). The total transitions vs total transversions, silent transitions vs silent transversions and replacement transitions vs replacement transversions were compared using a T test. P values <0.05 were regarded as significant. (F) Assessment of the occurrence of typical AID hotspots WRC/GYW (W=A/T, R=A/G, Y=T/C), WA/TW, or hotspots WRC and/or WGCW for the CATSST RF clone of B005 (top) and the MALT RF clone of B007 (bottom). ANA, antinuclear antibody; AutoAb, autoantibody; CDR3, complementarity determining region 3; FWR, framework region; Ig, immunoglobulin; RF, rheumatoid factor; RTX, rituximab.

Before RTX intra-clonal diversification was similar between ANA and RF clones (figure 2E). RF MALT clones showed large intra-clonal diversity compared with other RF clones (figure 2E), suggesting a role for aberrant intra-clonal diversification and increased cell survival in MALT lymphomagenesis from RF clones. After RTX large non-MALT RF clones displayed increased intra-clonal diversification (figure 2E). To allow analysis of Ig expression at the individual cell level, Ig-sc-RNAseq was performed in the tissues, obtained freshly at relapse after RTX (online supplemental figures 1 and 2). In advantage to bulk Ig-RNAseq, sc-Ig-RNAseq allows a precise analysis of intra-clonal diversification, since many Ig reads are sequenced per cell. In addition, it allows parallel analysis of general RNA expression per cell. Ig-sc-RNAseq confirmed that in both patients the most expanded RF clones at relapse after RTX displayed extensive intra-clonal diversification (figure 3A, B). The intra-clonal diversity of ANA clones was unaltered after RTX (figure 2E). The largest ANA clone displayed little intra-clonal diversification (figure 3C). General sc-RNAseq showed that RF clones consisted of a mixture of memory B cells, plasma cells and proliferating germinal centre-like cells (figure 2F). In contrast, ANA clones mainly concerned plasma cells. Proliferating B cells, including RF and ANA B cells, could be discerned as a separate cluster in principal component analysis. These expressed proliferation markers, such as MKI67, CDK1 and CDC20. A proportion expressed AICDA and BCL6 indicative of T cell dependent activation. Mutation analysis confirmed AICDA mediation (figure 3E, F). Taken together, during regeneration of disease manifestations after therapeutic B cell depletion, both MALT and non-MALT RF clones can regenerate as large clones in affected tissues and show continuous affinity maturation, accompanied by marked intra-clonal diversification.

### ANA clones display signs of similar antigen-driven affinity maturation that differs from RF clones

We analysed the antigen dependence of affinity maturation in ANA and RF clones using the BASEline tool (see online supplemental methods). Analysis of SHM patterns showed that all autoreactive clones displayed a similar antigen-dependent selection pressure (figure 3D). However, RF clones displayed a stronger negative selection pressure on the framework region (FR) compared with ANA clones, indicating that structural integrity of the variable gene segments is most important for positive selection of RF clones.

In an earlier study, selection of recombinantly expressed Igs from SjS tissues was enhanced by N-glycosylation sites in the B cell receptor variable region FR1, resulting in activation by C-type lectins.^16^ Few ANA and RF clones had N-glycosylation sites in FR1 (0%–10%). Most were present in FR3 or CDR2 without clear-cut differences in number between AutoAb specificities.

### Potential clinical utility of Ig-seq to assist lymphoma diagnosis

Finally, we analysed the potential utility of Ig-seq to assist lymphoma diagnosis in patients with SjS. For this, Ig clonality assessment was performed in affected tissues of two additional patients with SjS (B012 and B013), who had been referred from other hospitals because of challenging lymphoma diagnostics (detailed case descriptions in online supplemental methods). In both cases Ig-seq helped to establish a diagnosis of MALT lymphoma.

In B012, Ig-DNA-seq showed a monoclonal stereotypic RF expansion in biopsies of a liver and lymph node that had earlier received a diagnosis of primary biliary cirrhosis and autoimmune inflammation. Of interest, the same clonotype as in the lymphoma was detected in the top 40 of most abundant clonotypes present in a labial biopsy that had been performed for SjS diagnosis 2 years earlier before lymphoma onset.

In B013, Ig-seq showed that two suspected mass lesions in different tissues consisted of two different MALT lymphomas

## Discussion

This study shows that RF and ANA B cells are enriched in affected tissues of patients with SjS, where they occur as a small part of a polyclonal repertoire. RF and ANA clones affinity maturate in divergent fashion, which increases in patients with secondary RF lymphomagenesis. The RF repertoire displays IgM and antigen-dependent affinity maturation that coincides with intra-clonal diversification associated lymphomagenesis. Regeneration of clinical disease manifestations after RTX coincides with large RF clones, which not necessarily belong to the lymphoma clone, that display continuous affinity maturation and intra-clonal diversification.

In SjS, experimental and translational studies have suggested that autoreactive clones are generated from disturbed circulating B cell populations. Circulating naïve B cells in patients with SjS are enriched for polyreactive and nuclear antigen-reactive cells and circulating memory B cells contain anergic autoreactive clones.^16 29–31^ Here, we show that circulating memory B cell populations contain expanded B cell clones. However, the autoreactive repertoire is not associated with a restricted repertoire in a circulating B cell population. Instead, it is enriched in affected tissues, displaying antigen-dependent affinity maturation that associates with a preference for shared sequence motifs.

The clones producing Ig against nuclear proteins Ro60, Ro52 and La displayed features consistent with antigen-dependent selection of high Ig affinity clones: they were mostly small plasma cell clones, consistently expressed IgG1, had a relatively high SHM load and exhibited limited intra-clonal diversification. A proportion of ANA clones precipitated with multiple antinuclear antigens, suggesting a polyreactive nature. The large number of ANA clones displaying similar affinity maturation suggests that these evolved as a result of intramolecular and intermolecular epitope spreading. In contrast, RF clones displayed features of suboptimal affinity maturation: they occurred less often, expressed IgM, had a relatively low SHM load and depended on specific variable gene segments. After RTX, the SHM load and the intra-clonal diversification of the most dominant RF clones increased. This supports continuing affinity maturation of RF clones.

The predominant expression of IgM by RF clones versus IgG1 by ANA clones in the context of a similar antigen-dependent selection pressure suggests that their isotype use determines their susceptibility to lymphomagenesis. Class-switching to IgG1 induces a preference toward plasma cell differentiation.^32^ The continued expression of IgM by all detectable RF clones may be caused by the dependence of optimal RF B cell receptor stimulation by immune complexes that can cross-link multiple IgM isotype RF BCRs on the clonal membrane.^33^ Such a required stereochemistry to pass the threshold for sufficient B cell receptor activation would explain the observed selection dependence on the use and structural integrity of a restricted set of Ig variable regions. Continued expression of IgM, together with less access to co-stimulatory signals, may result in suboptimal affinity maturation. A proportion of RF clones exhibited continuing proliferation and extensive intra-clonal diversification. These observations are in line with gradual accumulation of lymphoma driver mutations in germinal-centre-like cells.^10^

In both patients that were treated with RTX, clinical relapse coincided with expansion of autoreactive clones. The extensive depletion of B cells after RTX induces a reciprocal increase in B cell activating factor (BAFF) levels, which may facilitate clonal expansion of new and persisting clones.^34 35^ In mice induced with a T–B cell dependent form of experimental encephalomyelitis, part of autoreactive memory B cells persisted after RTX in lymphoid tissue and disease flare was associated with expansion of these cells within a restricted repertoire.^36^ The observed increase in SHM load of dominant RF clones after RTX may be explained by increased positive selection of RF clones because of increased BAFF levels. Moreover, in both patients, RF clones proliferated in the affected tissues at relapse after RTX. This was the solitary lymphoma clone in B007 and multiple non-lymphoma RF clones in B005. Intriguingly, B007 experienced a prolonged clinical response of 2 years, while in B005 the disease relapsed within 6 months. In patient, B005 new RF clones proliferated quickly after RTX and likely contributed to the increase in SG swelling after 6 months.

Finally, from a clinical perspective, we found that RTX did not succeed in abrogating lymphomatous B cell clones. Possibly, other B cell depletive treatments may have superior efficacy. Besides this, it can be challenging to discriminate MALT lymphoma from SjS-associated inflammation, determine the best treatment regimen and assess response to treatment. A diagnosis is made by assessing the combination of clinical presentation, histology, phenotype and sometimes clonality analysis and/or genetic studies. Also in the study patients, the diagnosis of MALT lymphoma had been challenging. Ig-seq retrospectively could have assisted in establishing a diagnosis earlier and more precisely. Future prospective investigations should investigate in more patients in more detail the added value of Ig-seq for diagnostic problems in patients with SjS with one or more mass lesions and for detection of small (pre-)lymphomatous clones in major SGs.

To summarise, we used for the first time an integrated omics workflow to analyse the generation of the autoreactive repertoire in circulating B cell populations and affected tissues of patients with SjS, and demonstrated tissue restricted, aberrant affinity maturation of RF clones compared with ANA clones in inflamed tissues. These data give insight into the molecular mechanisms of autoreactive inflammation and MALT lymphoma, and help to analyse the clinical response to RTX treatment in individual patients.

10.1136/annrheumdis-2021-221604.supp9 Supplementary data

10.1136/annrheumdis-2021-221604.supp10 Supplementary data

10.1136/annrheumdis-2021-221604.supp11 Supplementary data

## Data Availability

Data are available in a public, open access repository. Single-cell FASTQ files were deposited at the NCBI as BioProject ID PRJNA742201 and PRJNA7883525.
