# Supplementary figures and images for: Proteogenomic analysis of the autoreactive B cell repertoire in blood and tissues of patients with Sjögren’s syndrome
Source: Ann Rheum Dis. 2022 Feb 10;81(5):644–52. doi: 10.1136/annrheumdis-2021-221604 (PMC8995816; doi:10.1136/annrheumdis-2021-221604)

Ig analysis

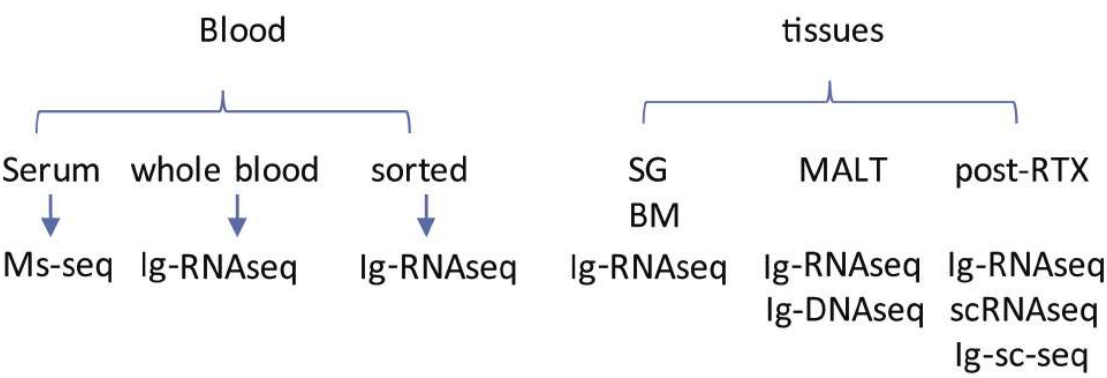

Supplement: Supplementary data [file annrheumdis-2021-221604supp001.pdf]

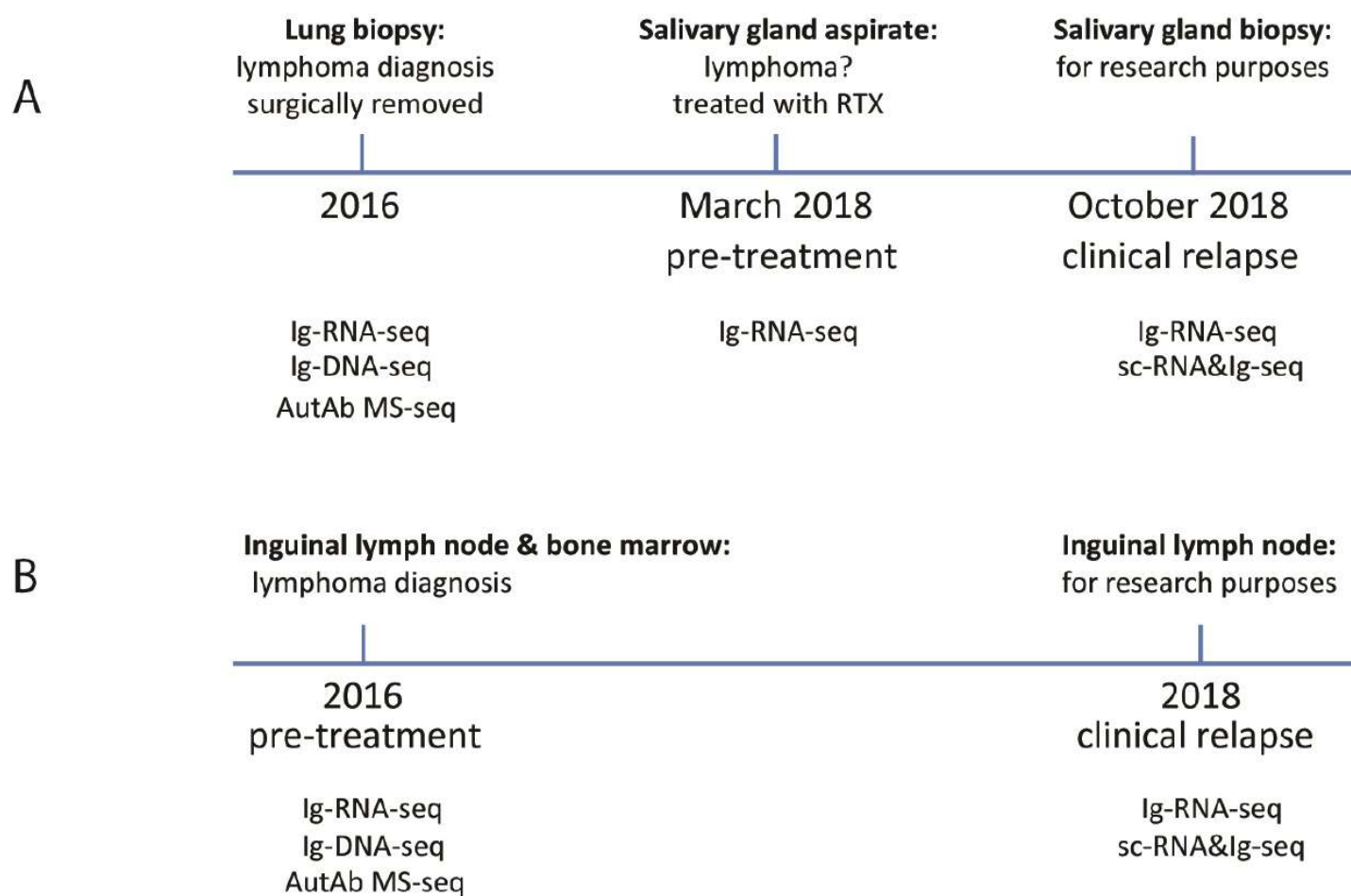

Supplement: Supplementary data [file annrheumdis-2021-221604supp002.pdf]

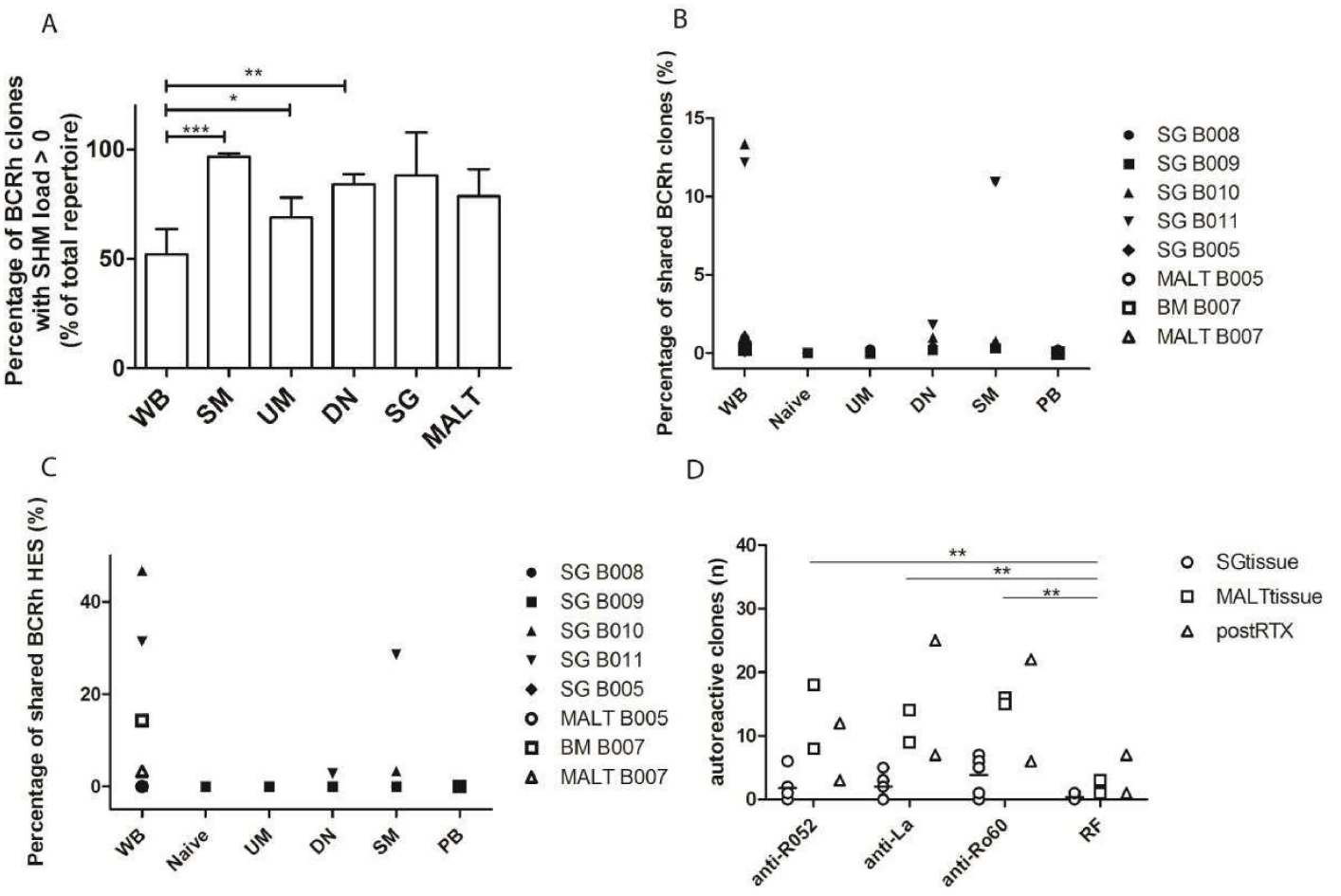

Supplement: Supplementary data [file annrheumdis-2021-221604supp004.pdf]

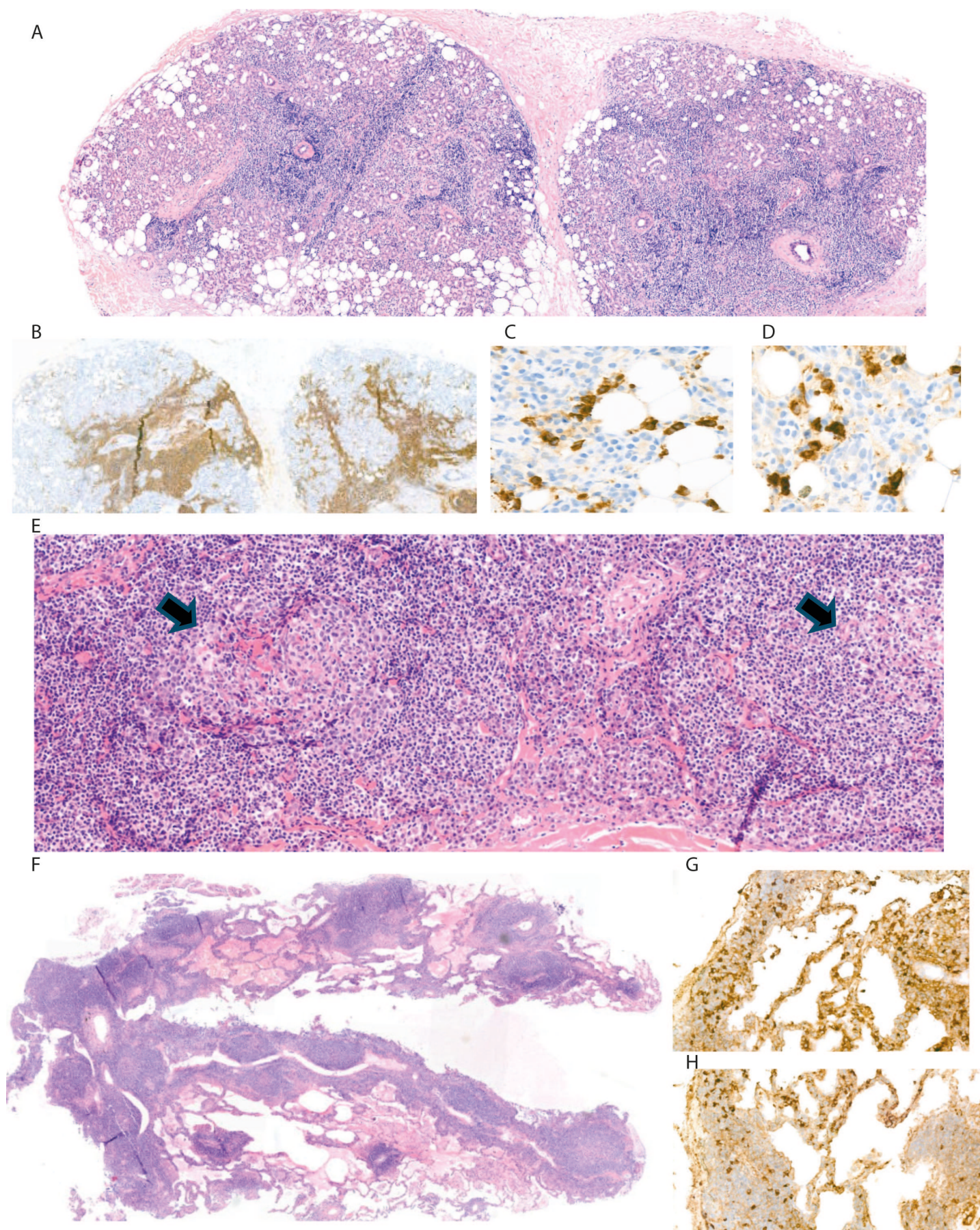

Supplement: Supplementary data [file annrheumdis-2021-221604supp003.pdf]

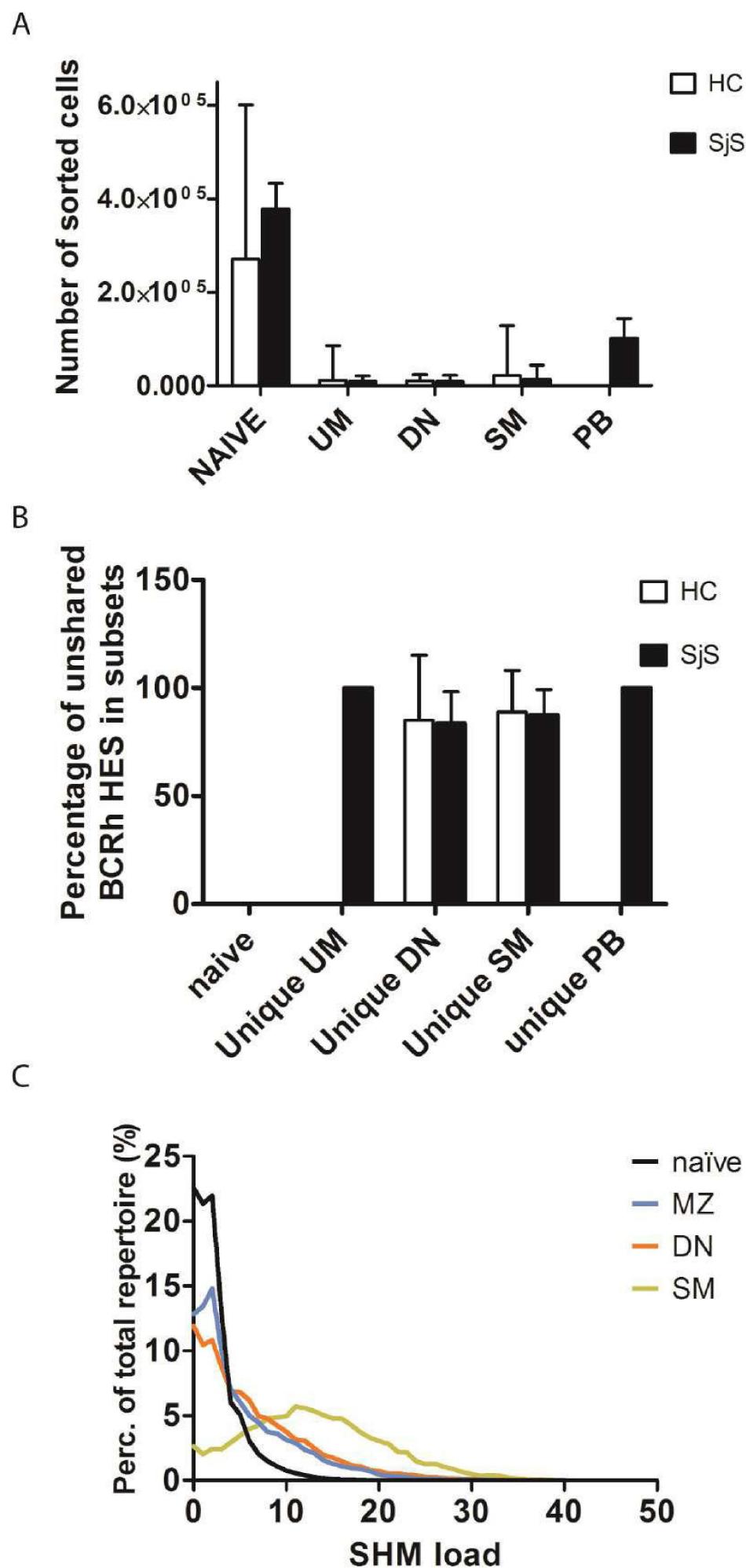

Supplement: Supplementary data [file annrheumdis-2021-221604supp005.pdf]

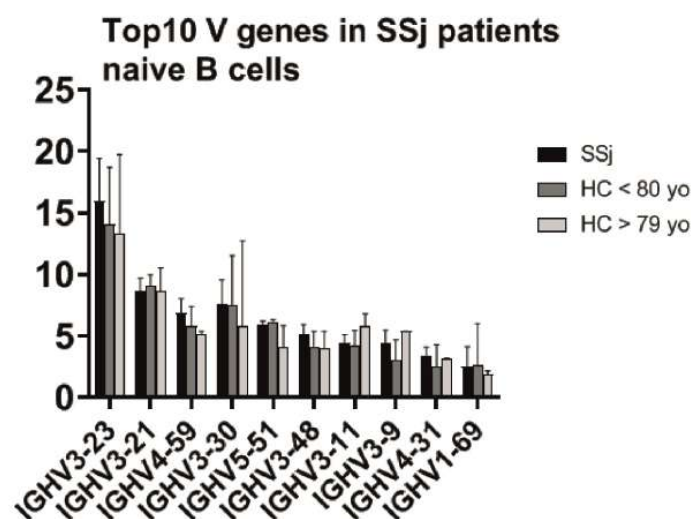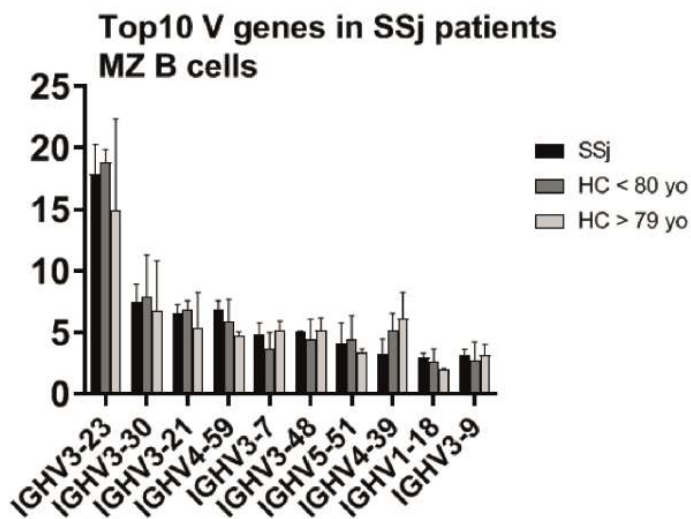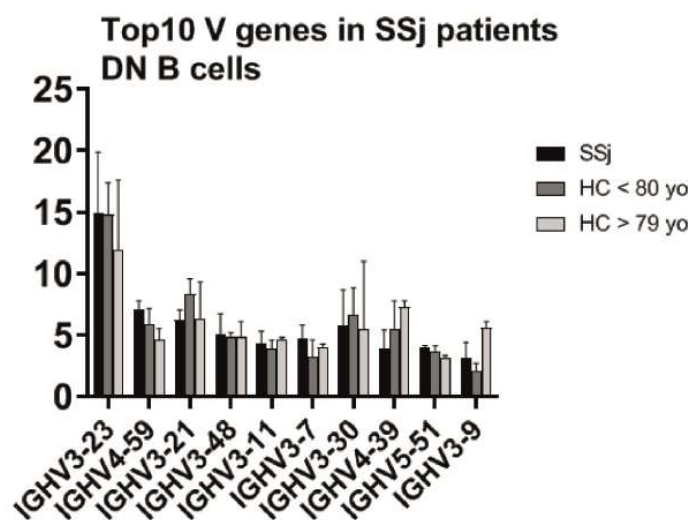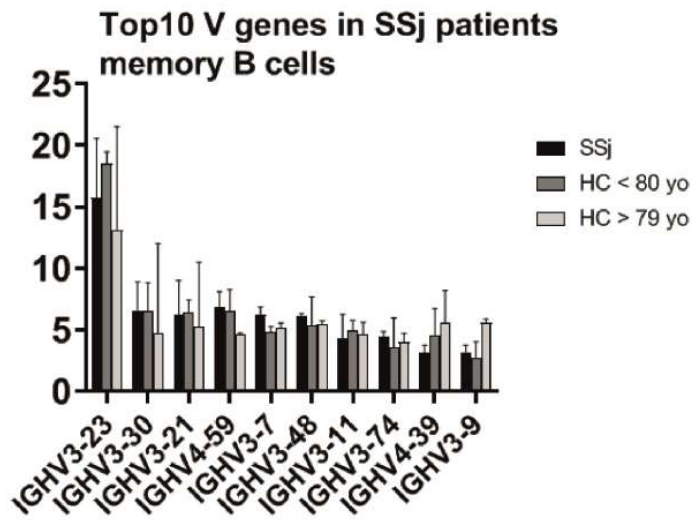

Supplement: Supplementary data [file annrheumdis-2021-221604supp007.pdf]

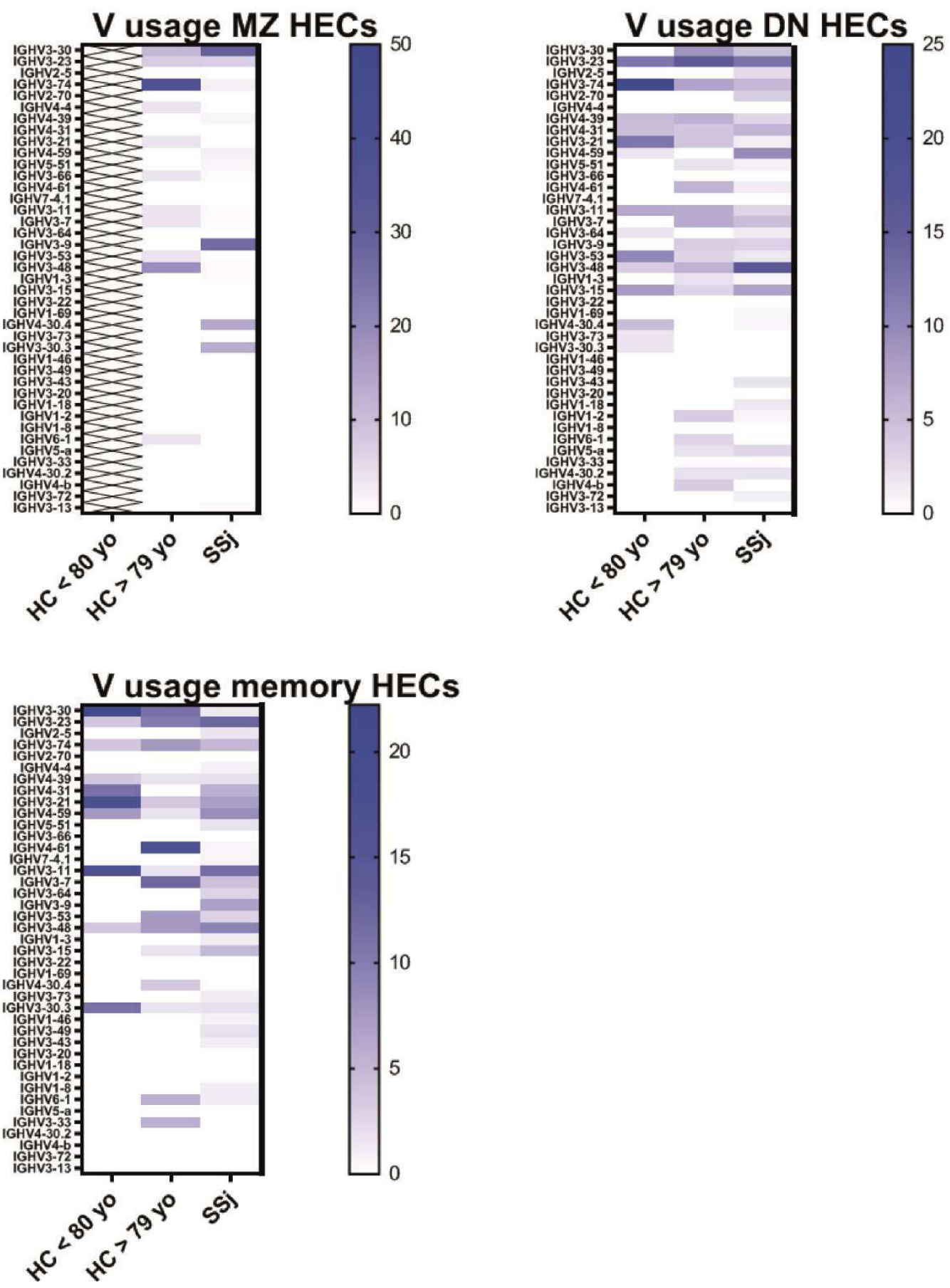

Supplement: Supplementary data [file annrheumdis-2021-221604supp008.pdf]

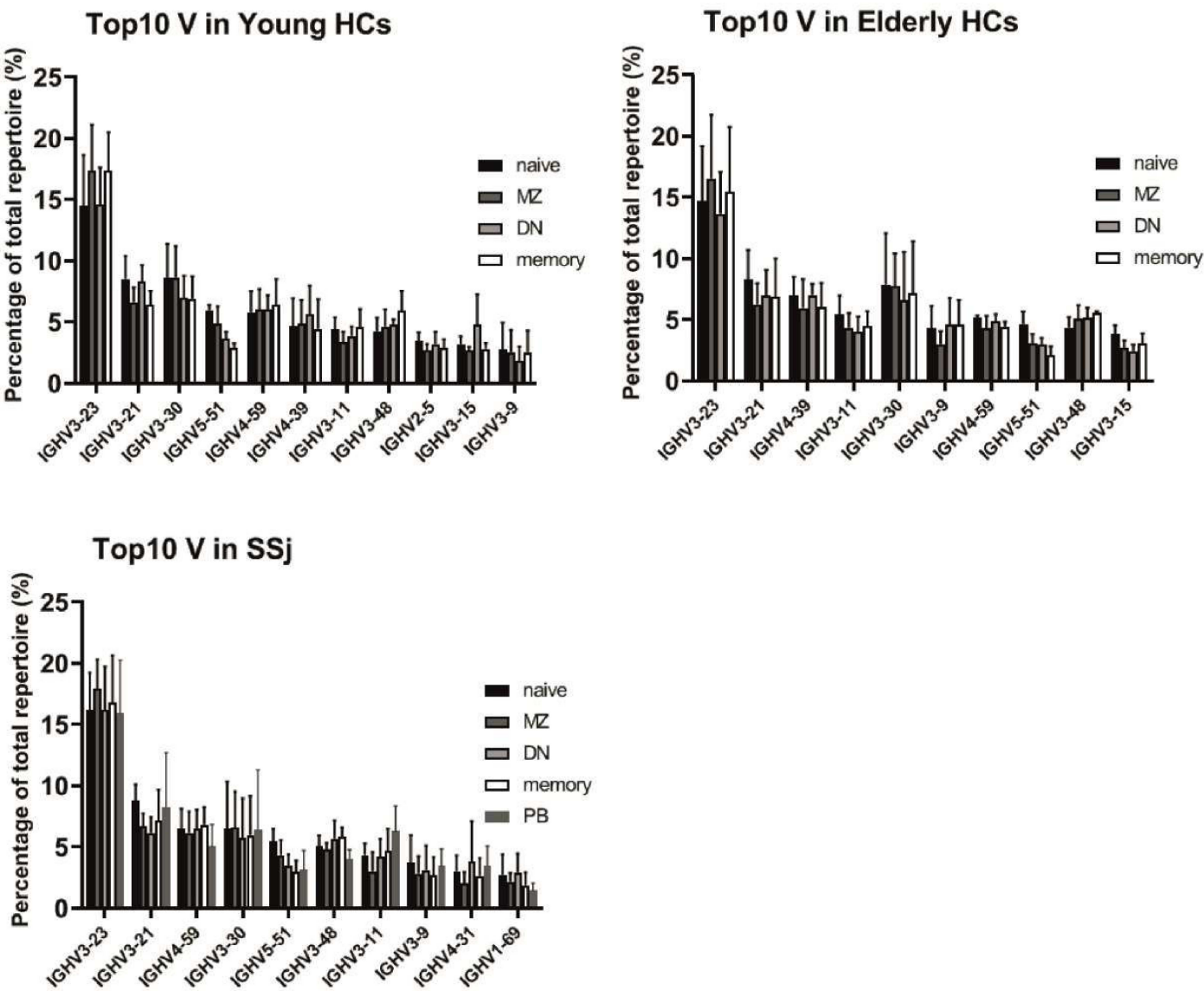

Supplement: Supplementary data [file annrheumdis-2021-221604supp006.pdf]

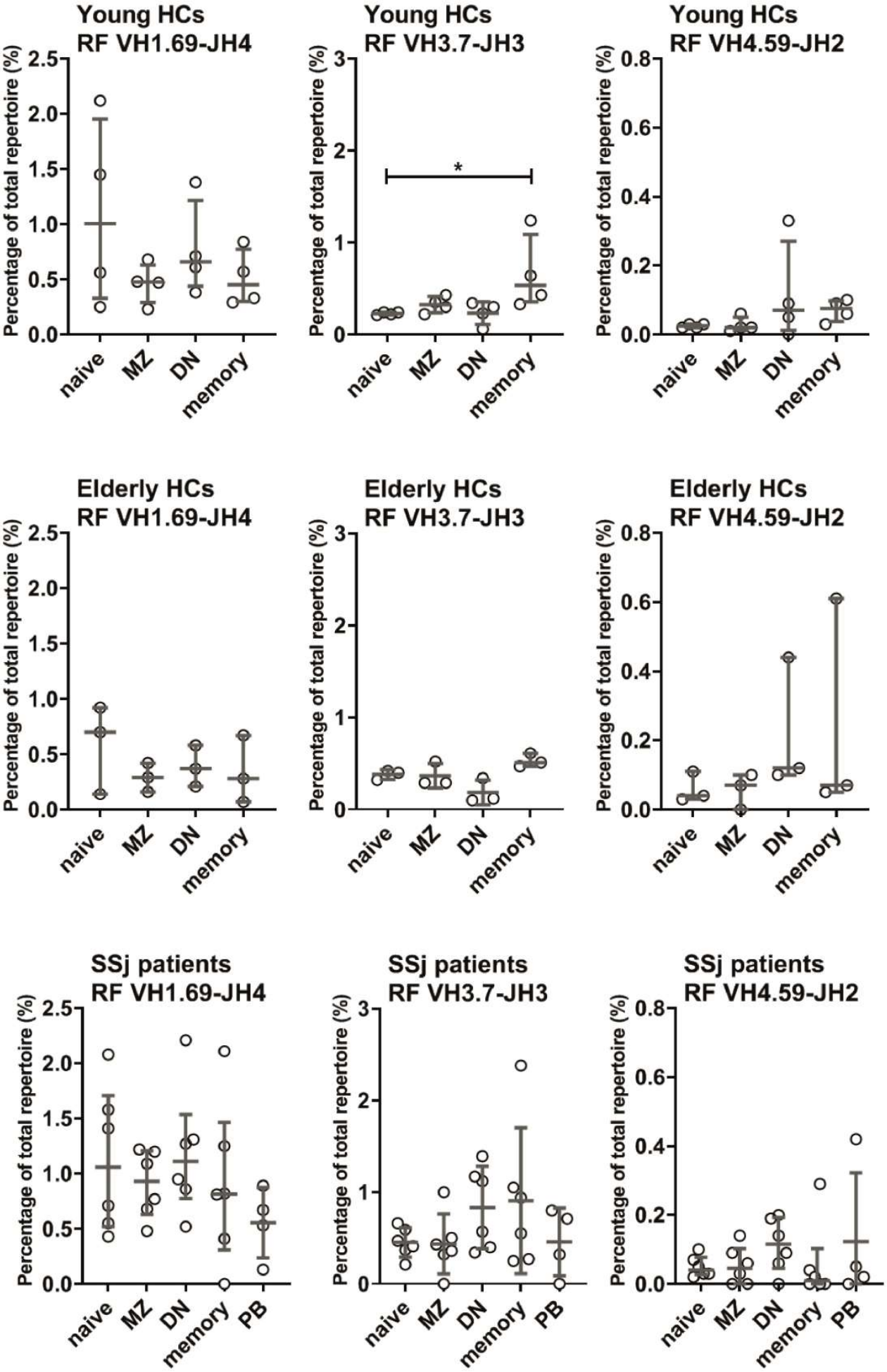

Supplement: Supplementary data [file annrheumdis-2021-221604supp009.pdf]

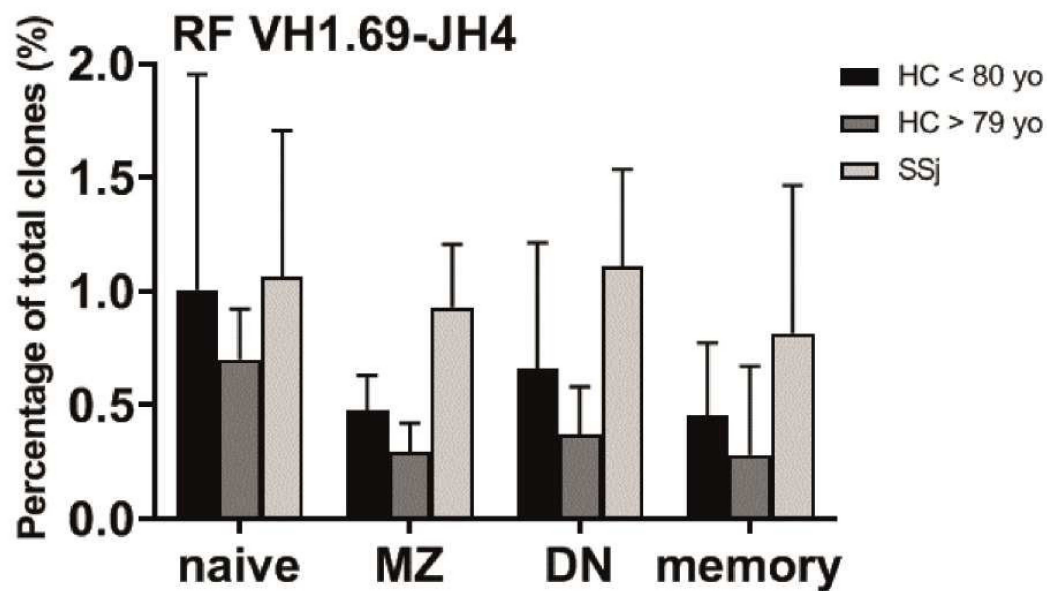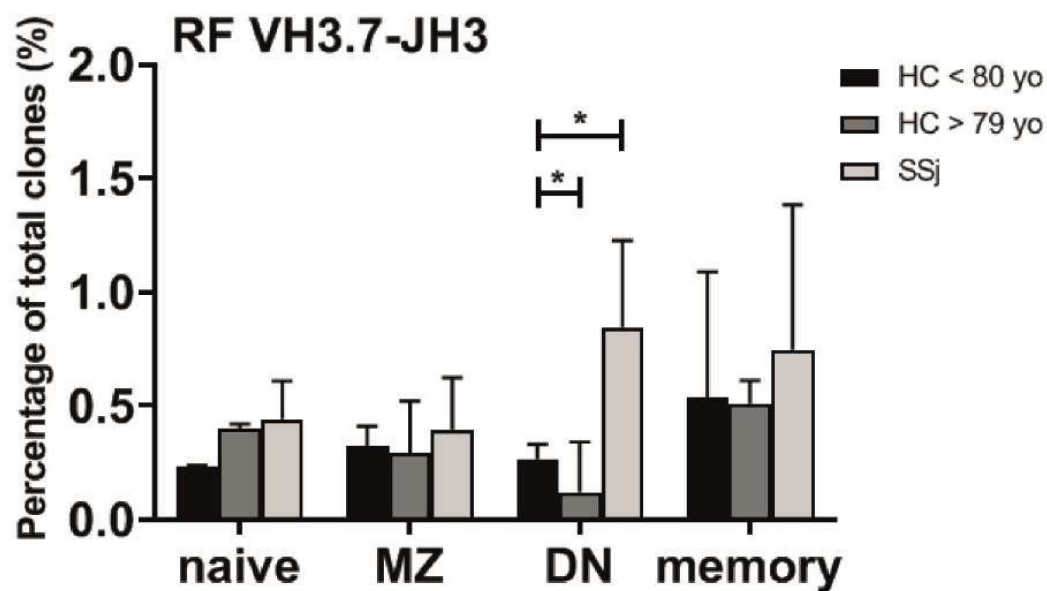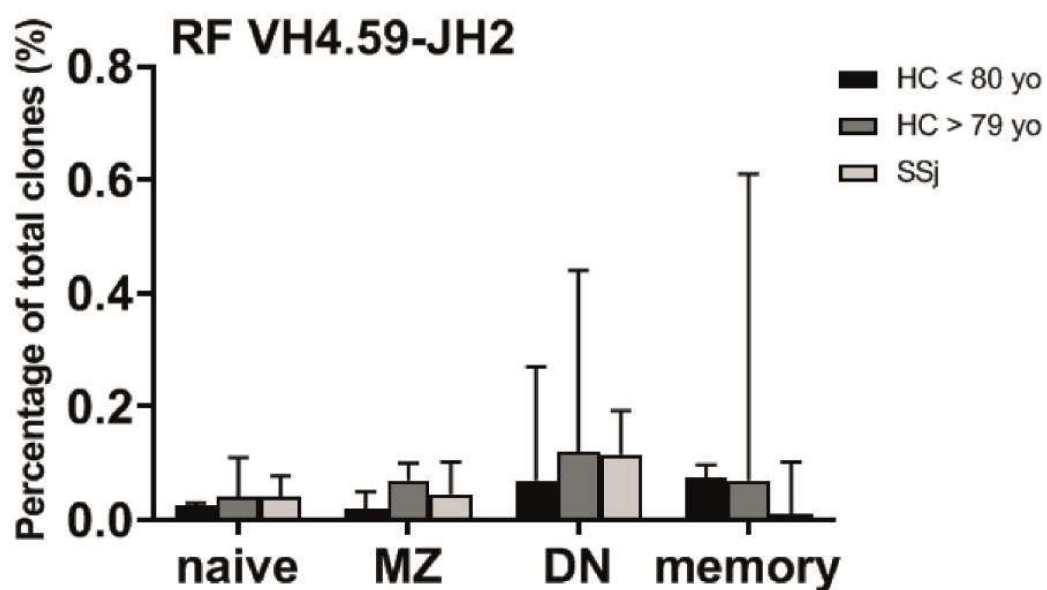

Supplement: Supplementary data [file annrheumdis-2021-221604supp010.pdf]

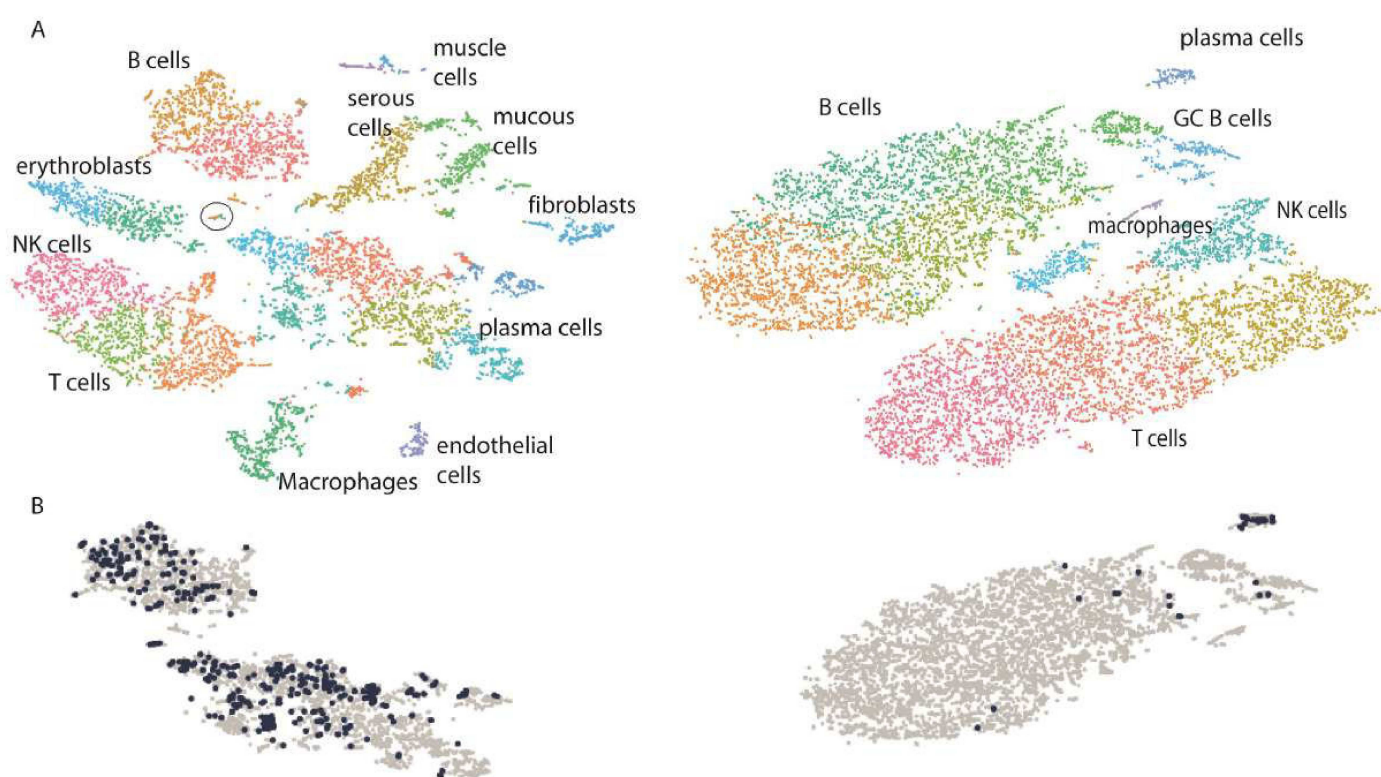

Supplement: Supplementary data [file annrheumdis-2021-221604supp011.pdf]
