# Supplementary material for: Proteogenomic analysis of the autoreactive B cell repertoire in blood and tissues of patients with Sjögren’s syndrome
Source: Ann Rheum Dis. 2022 Feb 10;81(5):644–52. doi: 10.1136/annrheumdis-2021-221604 (PMC8995816; doi:10.1136/annrheumdis-2021-221604)
Supplement: Supplementary data [file annrheumdis-2021-221604supp012.pdf]

Supplementary table 1

patient	PAXgene	memB	SG	BM	MALT	postRTX
B005	39104	46054	35335	-	20117	46742
B007	42586	-	-	22750	13145	35567
B008	26203	101442	1697	-	-	-
B009	20301	99705	1595	-	-	-
B010	14652	41805	26343	-	-	-
B011	17834	67752	899	-	-	-
HC1	34065	79093	-	-	-	-
HC2	76047	287083	-	-	-	-
HC3	54067	205419	-	-	-	-
HC4	72062	204501	-	-	-	-
HC5	35355	121727	-	-	-	-
HC6	31075	124629	-	-	-	-
HC7	57629	171264	-	-	-	-

Supplementary table 1. Number of Ig-RNAseq reads per sample.
PAXgene = whole blood samples; memB = pooled sorted memory B cell subsets; SG = salivary gland; BM =bone marrow; MALT = MALT lymphoma tissue. B005: lung excision, B007: excised lymph node. postRTX = tissue at disease relapse after rituximab. B005: lip biopsy, B007: lymph node biopsy.

Supplementary table 2

		CDR3	IGHV	IGHJ	IGVK/IGVL
**anti-Ro52**	also anti-Ro60	CARMGILGN	v1-18	j3	vk1-27
	also anti-Ro60	CARHLFGVV	v3-11	j4	vk3-20
	also anti-Ro60	CAREPILT	v3-48	j5	vk1d-39
	also anti-Ro60,anti-La	CAKGTPFSA	v3-23	j4	vk1-12
	also anti-Ro60	CAKDKNRFG	v3-30	j4	vl2-14
	also anti-La	CARLNYYDT	v4-59	j4	vk2d-28
**anti-La**	also anti-Ro60	CARDSLRAPP	v4-39	j4	vl3-1
	also anti-Ro52	CARLNYYDT	v4-59	j4	vk2d-28
	also anti-Ro52,anti-Ro60	CAKGTPFSA	v3-23	j4	vk1-12
**anti-Ro60**	also anti-Ro52	CARHLFGV	v3-11	j4	vk3-20
	also anti-Ro52	CARMGILGN	v1-18	j3	vk1-27
	also anti-Ro52	CAREPILT	v3-48	j5	vk1d-39
	also anti-Ro52,anti-La	CAKGTPFSA	v3-23	j4	vk1-12
	also anti-La	CARDSLRAPP	v4-39	j4	vl3-1
	also anti-Ro52	CAKDKNRFG	v3-30	j4	vl2-14

Supplementary table 2. Variable gene usage of Ig precipitating with multiple ANA in sc-Ig-RNAseq.
CDR3 = complementarity determining region 3, IGHV = immunoglobulin heavy chain variable gene segment, IGHJ = immunoglobulin heavy chain joining gene segment, IGVK/L = immunoglobulin kappa/lambda light chain variable gene segment usage.

SUPPLEMENTAL METHODS

Patients

All patients had tested positive for anti-Ro, anti-La/SSB (hereafter referred to as anti-La) and RF autoantibodies during diagnostic evaluation. Presence of autoreactive B cell clones was analyzed in blood versus various tissues. In 5 patients salivary gland tissue was available for analysis. Additional tissues were analyzed in 2 of the 6 patients (patient B005 and B007) with a diagnosis of MALT lymphoma. The lymphoma tissue of patient B005 concerned excised lung tissue, the tissue of B007 an excised inguinal lymph node. For B005 salivary gland tissue was available for comparative analysis. These 2 patients were treated with RTX and the autoreactive clones in salivary gland (B005) and inguinal lymph node tissue (B007) at relapse of clinical manifestations were studied.

Patient B005 was diagnosed with SjS in 1986. In 2016 a pulmonary mass was excised. Pathological examination revealed extensive areas of diffuse infiltration of lambda+ plasma cells with interspersed small nodular B-cell aggregates with limited BCL6+ germinal center formation and extensive vascular and interstitial amyloid deposition, leading to a diagnosis of MALT lymphoma. Oncological staging did not reveal other suspected sites and therefore the excision was considered sufficient treatment. There was no cryoglobulinemia nor signs of amyloid deposition in other organs. In 2018 this patient had increasing complaints of a unilateral parotid gland swelling that was deemed unlikely to be a lymphoma based on repeated MRI and ultrasound imaging. A biopsy was decided against because of high local neoangiogenesis. A parotid aspirate did not allow discrimination between inflammation and lymphoma. Patient was treated pragmatically with RTX and experienced a partial decrease in swelling that increased again after 6 months. After return of salivary gland swelling a labial gland biopsy was obtained for the study (no histology available), after which the patient was treated again with rituximab.
Patient B007 had a diagnosis of SjS since 2009, based on a labial biopsy, and developed cryoglobulinemic vasculitis in 2016. A PET scan revealed diffuse lymphadenopathy. A MALT lymphoma was diagnosed based on an excised inguinal lymph node. Pathological examination showed sheets of IgM/kappa-positive plasma cells. The patient was treated with RTX, after which complaints completely subsided for 2 years. When complaints returned in 2018 a biopsy of a juxtaposed earlier involved inguinal lymph node was performed for the study (no histology available), after which the patient was re-treated with rituximab. Cryoglobulinemia concurred with a highly elevated serum IgM-RF level of 4520 IE/mL that became 26 IE/mL with absent cryoglobulins after RTX treatment. In 2018 when complaints returned cryoglobulins also returned and the IgM-RF level increased again to 804 IU/L.

Patient B012 had a diagnosis of SjS, based on sicca complaints, a labial biopsy and anti-Ro60 and anti-La positivity. Two years later he developed night sweats and purpuric lesions on the legs. Diagnostic work-up in the referring hospital had shown liver enzyme abnormalities, a positive rheumatoid factor, cryoglobulins and diffuse lymphadenopathy. A liver biopsy had yielded a diagnosis of primary biliary cirrhosis. An inguinal lymph node biopsy had shown a monoclonal B cell population but had been decided to best fit with polyclonal B cell expansion in the context of an active autoimmune disease. Patient was referred because of

acute renal insufficiency of unknown cause, suspected to be caused by cryoglobulinemic vasculitis or lymphoma. Upon re-examination of the biopsies and NGS-based Ig rearrangement analysis a diagnosis of MALT lymphoma was made in both the liver and lymph node biopsies. Immunohistochemistry showed CD20 positive B cell aggregates with few kappa positive plasma cells the liver and B cell aggregates with many kappa positive plasma cells in the lymph node. Ig-DNAseq showed a large monoclonal expansion in both tissues using IGHV1-69-IGKV3-20, similar to those used by stereotypic RFs. In the lip biopsy, that had been acquired for diagnosis 2 years earlier, the clonotype of the MALT lymphoma was detected in the most abundant clonotypes, which might indicate the presence of a precursor clone in a polyclonal infiltrate. The diagnosis was revised into MALT lymphoma with cryoglobulinemic vasculitis, probably caused by a stereotypic RF.

Patient B013 had a diagnosis of SjS, based on sicca complaints, parotid swelling, arthralgias, fevers and lymphadenopathy. Anti-nuclear antibodies and cryoglobulins were detected, anti-Ro60/anti-Ro52/anti-La and RF were negative. Diagnostic work-up in the referring hospital had shown a pulmonary mass lesion and a subcutaneous lesion in the right mamma. A diagnosis of disseminated MALT lymphoma had been suspected, based on a transbronchial biopsy of pulmonary tissue and a biopsy of the subcutaneous tissue. Pathological re-examination of the subcutaneous tissue showed nodular CD20 positive B cell aggregates, kappa positive plasma cells with CD23/CD21 positive germinal center remnants. The pulmonary tissue biopsy showed nodular and monocytoid CD20 positive B cell aggregates and plasma cells without light chain restriction. Ig-clonality assessment showed 2 different monoclonal expansions in these biopsies. The amplicon sizes and therefore the sequences of the clonal rearrangements detected in these expansions differed. Both expansions had a rearrangement of the IGK-DE gene, but the amplicon-size differed. The subcutaneous expansion also had a rearranged IGH-DJ sequence that did not occur in the pulmonary expansion. The diagnosis was revised into 2 concurrent MALT lymphomas.

Collection of blood samples and fluorescence-assisted cell sorting (FACS)
Whole blood was collected for Ig-RNAseq in PAXgene tubes (Qiagen, Venlo, the Netherlands) at the day of tissue biopsy and stored at -80°C until further processing. For B cell subset sorting, up to 5 10 ml VACUETTE® or heparin tubes (Greiner Bio-One, Alphen a/d Rijn, the Netherlands) were filled with patient blood and inverted. Subsequently, the PBMCs were isolated by a density gradient using Lymphoprep™ (Stem Cell Technologies, Cologne, Germany) according to the manufacturer's recommendations, including a 20 minute spin at 800g. Cells were resuspended at 10^7 cells/ml and stained with a 1:100 dilution of αIgD-FITC, αCD27-Pe-Cy5.5, αCD38-PC7, αCD19-APC-AF750 for memory B cell subset sorts or stained with αCD138-FITC, αCD3-PE, αCD38-PC7 and αCD20-PB for plasmablast sorts. All FACS sorts were performed on the FACS Aria (BD Biosciences, San Jose, CA, USA). Memory B cells were first gated for single-cell live $CD19^+CD38^-$ cells. Subsequently, IgD^-CD27^- were regarded as double-negative (DN), naïve B cells were IgD^+CD27^-, switched memory (SM) were IgD^-CD27^+ and unswitched memory (UM) B cells were IgD^+CD27^+. Plasmablasts (PB) were gated as $CD3^-CD20^-CD38^+CD138^-$. The numbers of sorted cells are depicted in Supplementary Figure 4A. Sorted fractions were collected in cold RLT lysis buffer (Qiagen, Venlo, the Netherlands) supplemented with 1% β-mercaptoethanol (Sigma-Aldrich, Zwijndrecht, The Netherlands) and subsequently snap-frozen and stored at -80°C until further processing.

Processing of tissue samples

Tissue samples were acquired by ultrasound-guided biopsy, with the exception of the 2018 pre-treatment parotid gland of B005, which was acquired by fine-needle aspiration, the lung biopsy of B005 and the 2016 lymph node biopsy of B007, which were surgically removed. Tissue samples were processed in 3 ways: For bulk Ig RNA-seq, samples were frozen in Tissue-Tek O.C.T. compound (Sakura, Alphen a/d Rijn, the Netherlands) or formalin-fixed and paraffin-embedded (FFPE). For single-cell immunoglobulin expression, tissue samples were processed immediately. To prepare single-cell suspensions, lymph node biopsies were squeezed through a 70 µm pore cell strainer (Corning, NY, USA) 3 times. Red blood cells (RBCs) were lysed for 2 min at room temperature (RT) in 4 ml RBC lysis buffer (155 nM NH_4Cl, 12 mM $KHCO_3$, 0.1 mM EDTA, pH 7.3). Salivary gland tissue was digested using Liberase™ (Roche, Basel, Switzerland) for 1h at 37°C prior to cell straining and RBC lysis.

Immunohistochemistry was performed during clinical work-up according to standard protocols for CD20 (clone L26, Thermo Fisher Scientific, Landsmeer, Netherlands), CD79a (), kappa (polyclonal, DAKO, Agilent , Amstelveen, Netherlands) and lambda (polyclonal, DAKO, Agilent , Amstelveen, Netherlands).

Pretreatment of samples for Ig-RNA sequencing

The cryovials containing samples stored in freezing medium were placed in a water bath at 37°C until nearly completely thawed. The cell suspensions were placed in 50mL Falcon tubes and 16 mL of cold Thawing Medium (IMDM with 20% FCS, 0.00036% (v/v) β-mercaptoethanol and penicillin-streptomycin) were added slowly. Samples were stored 20 minutes at room temperature in the dark and then centrifuged for 10 minutes at 1700 rpm. After centrifugation, the supernatant was removed and the pellets were resuspended in 25 mL of room temperature Washing Medium (RPMI with 4% FCS, 0.025M Tris-HCl and penicillin-streptomycin). After centrifugation for 10 min at 1700 rpm, the supernatant was removed and the pellets were resuspended in 600 µL RLT lysis buffer (Qiagen) supplemented with 1% β-mercaptoethanol (Sigma-Aldrich).

RNA extraction for next-generation sequencing

For the peripheral blood samples, RNA extraction was performed using the PAXgene Blood RNA Kit (Qiagen) on the QIAcube instrument (Qiagen) according to the manufacturer’s instructions. For all the cells in lysis buffer, 10,000 non-BCR-expressing HEK293T cells were added prior to RNA extraction to improve the efficiency of the procedure. RNA extraction was performed using the RNeasy Mini Kit (Qiagen) or the RNeasy Micro Kit (Qiagen) depending on the initial number of cells in the samples, according to the manufacturer’s instructions. For the FFPE tissue sections, miRNeasy FFPE Kit (Qiagen) was used, followed by RNeasy Micro Kit (Qiagen), with the following adaptation: 20 µL of 10,000 HEK293T cells lysate were added to the samples prior to DNase treatment. For all samples, RNA was eluted in RNase-free water (80 µL elution volume for blood samples, 30 µL for the RNeasy Mini Kit samples and 16 µL for the RNeasy Micro Kit samples) and its concentration was measured at the NanoDrop 2000/2000c spectrophotometer (Thermo Fisher Scientific, Breda, The Netherlands).

RNA-based next-generation sequencing of Ig repertoires

Using 125 ng of the obtained RNA, a specific complementary DNA (specific-cDNA) of BCRh molecules was synthesized using a BCR heavy-chain Constant region reverse primer tagged

with a 9 random nucleotide unique molecular identifier (UMI) and a consensus sequence. The reaction using SuperScript III Reverse Transcriptase (Thermo Fisher Scientific) was performed according to the manufacturer's instruction for cDNA synthesis with custom primers. After specific-cDNA synthesis, Exonuclease I (Thermo Fisher Scientific, Breda, The Netherlands) treatment was performed according to the manufacturer's instruction to ensure left over primers removal. Ten µL of obtained product were then used in a Multiplex PCR with 23 forward primers covering all BCR heavy-chain Variable genes in combination with a reverse primer binding to the consensus sequence previously introduced in the specific-cDNA and tagged with an 8 bp patient identifier (MID, Molecular Identifier). Both forward and reverse Multiplex PCR primers were respectively tagged at the 5' end with Read 2 and Read 1 primers from the MiSeq system (Illumina). The Multiplex PCR was performed as follows: initial denaturation at 96 °C for 15 minutes, 35 amplification cycles (96 °C for 30 s, 60 °C for 1 min, 72 °C for 30 s), final elongation step at 72 °C for 10 minutes. Obtained products were purified with two rounds of AMPure XP beads (Beckman Coulter) in a 1:1 ratio. Purified products were then quantified using Qubit dsDNA HS Assay Kit (Thermo Fisher Scientific) and 50 ng were used for the PCR with the Nextera i7 and i5 Index primers according to the manufacturer's instructions (Illumina). The obtained amplicons are purified with two rounds of AMPure XP beads (Beckman Coulter) in a 1:1 ratio, quantified using Qubit dsDNA HS Assay Kit (Thermo Fisher Scientific), mixed according to the desired number of reads and sequenced using the Illumina Miseq Kit v3 2 x 300 bp technology according to the manufacturer's manual (Illumina, San Diego, California, USA). All primers used were ordered from Biolegio, Nijmegen, The Netherlands. Data were deposited at the NCBI as BioProject ID PRJNA788352[1].

RNA-based Ig repertoire analysis

The obtained sequencing reads were analyzed with an in-house developed workflow called RESEDA, available at https://bitbucket.org/barbera/reseda. The paired-end sequences were assembled using PEAR[2] and aligned to the IMGT gene database[3] with BWA (default settings)[4]. Variants were called with samtools mpileup and VarScan (the minimum coverage was set to 1 read)[5 6]. All differences with respect to the IMGT sequences were therefore considered as somatic hypermutations. The CDR3 sequences were determined by translating the sequences to peptide sequences and searching for conserved motifs in the V and J genes. We searched for N-glycosylation sites using the motif N-X-S/T (where X is any amino acid except proline). A list of clones was obtained by grouping the sequences by their unique V and J name and CDR3-nucleotide sequence [reseda]. We calculated the sum/average/mode number of mutations and N-glycosylation sites per subclone and the average percentage of mutations in comparison to the sequence length. The obtained Ig reads were analyzed using custom scripts in R studio (R version 3.3.2). The absolute number of mRNA molecules was determined by the number of unique UMIs in the sample, hereafter simply referred to as reads. The frequency of each clone was calculated as percentage of the total reads. Sequences with a frequency ≥ 0.5% were defined as highly expressed sequences (HESs), based on previous observations [7]. As a quality control for the sorting strategy of circulating memory B cell subsets the presence of and overlap in HESs was compared between sorted subsets. In none of the analyzed individuals HESs occurred in IgD+CD27- naïve B cells (Suppl. Fig. 4B). The majority of HESs were only expanded in one memory subset and no sequences in memory B cell HESs were shared with sorted plasmablasts. The somatic hypermutation (SHM) load, number of N-glycosylation sites, use of VJ segments and

RF associated stereotypes (using the same variable heavy and light chain gene segments, irrespective of complementary region sequence), were assessed for each Ig read. As a quality control for the protocol to assess SHM load, subsets were compared within samples. This showed an increasing SHM load between naïve, UM, SM and DN B cell subsets for each sample (Suppl. Fig. 4C). For analysis of intra-clonal diversification the number of clonotypes was analyzed. Clonotypes were defined as reads with a maximum of 2 mutation in the CDR3 on nucleotide level and an identical heavy chain VDJ rearrangement.

DNA-based next-generation sequencing of Ig repertoires
DNA extraction from FFPE samples was performed using the QIAamp DNA FFPE Tissue Kit (Qiagen) followed by QIAamp DNA Mini Kit (Qiagen). DNA quality was assessed as described previously [8]. The complete protocol for the NGS of genomic immunoglobulin heavy and light chain rearrangements on FFPE samples was described earlier [8]. In brief, three Multiplex PCRs were carried out on 40 ng of DNA each in order to obtain IGHV-IGHD-IGHJ-FR3, IGHD-IGHJ, IGKV-IGKJ and IGKV/Intron-KDE amplicons that were subsequently processed for Ion Torrent sequencing. The bioinformatics data analyses were carried out using ARResT/Interrogate (version 0.25.001) as previously described [8 9 10]. The obtained NGS-clonality data in ARResT/Interrogate were evaluated by pattern recognition in the context of the guidelines for interpretation and reporting of conventional Ig clonality assessment [3]. Sequencing data will be shared in a public repository upon acceptance of the manuscript.

Single-cell RNA sequencing of Igs
Single-cell immune profiling (Ig sequencing and gene expression analysis) was performed on salivary gland biopsy of B005 and the lymph node biopsy of B007 in duplicate and later combined. The Chromium single-cell immune profiling pipeline (10x Genomics, Leiden, the Netherlands) was used with the Chromium™ Single cell 5'Library &Gel Bead Kit and the Chromium™ Single Cell V(D)J Enrichment Kit, based on the manufacturer's protocol. 17,800 cells were used in each reaction to obtain a target of ~10,000 cells per analysis. Samples were sequenced on the Illumina NextSeq 500 with NextSeq 500/550 V2.5 kits for 150 and 300 cycles (Illumina). The 4 sequenced mRNA libraries displayed an average of 2095 ± 2813 reads/cell and an average of 678 ± 399 expressed genes/cell. Single-cell FASTQ files were deposited at the NCBI as BioProject ID PRJNA742201[11].

Single-cell RNA and Ig-Seq bioinformatics analysis
The resulting BCL files were processed using the CellRanger software pipeline v6.0.0 and analysed separately and combined with the Loupe Cell Browser V3.0.1 and Loupe VDJ browser V3.0.0 (10x Genomics). Biological replicates of B005 and B007 were combined for the analysis using cellranger aggr for the gene expression files and a manual combination of the Ig sequence files at the fastq level. T-distributed Stochastic Neighbor Embedding (t-SNE) projections were created with CellRanger and Loupe Cell Browser with default parameters. As an extra step to discard doublet cells from the B cell clusters cells were filtered that displayed unique genes of other cell populations: CD3E, VWF, C1QB and HBB. The plasma cell cluster was identified by CD79A, SDC1 and XBP1 expression, the memory B cell cluster by CD79A and CD27 expression and the GC cluster by CD79A, MKI67, BCL6, MEF2B and AICDA expression.

Custom extraction of CDR3s from single-cell RNA-seq data

Single-cell RNAseq reads that could not produce a complete heavy or light chain sequence were discarded by the 10x software, but the CDR3 information could still be present in the reads. Therefore, we applied the CDR3 identification step of the RESEDA workflow to the 10x data to obtain additional CDR3 sequences to include in the Ig reference library for autoantibody mapping. In brief, after translation to amino acid sequences in all possible reading frames, sequences were examined for conserved motifs in the V and J region, determined from a multiple sequence alignment of all V and all J genes provided by ImMunoGeneTics (IMGT) database [12]. Since the read pairs were not always overlapping, this CDR3 search was performed on both ends separately.

Analysis of Ig sequences

Cellranger output was combined with scripts from the Immcantation analysis framework (https://immcantation.readthedocs.io/en/stable/) using the stable version available on August 6, 2019. We used Change-O [13], Alakazam [14] and SHazaM [15] packages. Change-O allows parsing of 10x V(D)J output data and adds Immcantation-style annotation to the Cellranger output files, including alignment of full Ig sequences to the IMGT reference. After Ig alignment, the overall and nearest distance distributions were computed amongst all Igs per sample. These distributions, based on Levenshtein distance were used to infer an upper threshold for clonal groups, similar to the *findThreshold()* function from the SHazaM package. This threshold was used as an input to the DefineClones script from the Change-O package which assigned all Ig sequences to phylogenetic clones. This was done for heavy, lambda and kappa chains independently. Cells with Ig sequences containing an identical heavy chain VDJ assignment, an identical light chain VJ assignment and a maximum of 2 mutations at the CDR3 region on nucleotide level were regarded to be a clonotype. The selection pressure on the Ig sequences was calculated using the BASELINe tool [16]. The CDR3 regions were omitted in this analysis, because they contained many variations from the germline that were likely introduced during recombination. The occurrence of hotspot mutations was assessed manually. The BASELINe tool approximates antigen-dependent selection by examining the selection pressure based on the ratio of silent and replacement mutations of the Ig CDRs and framework regions (FWRs). In antigen-dependent B cell selection, the selection pressure on the Ig framework regions is often negative to maintain the structural integrity of the antibody and the selection strength on the CDRs is often positive or neutral.

Phylogenetic tree construction

The resulting clonal assignments per Ig (and all other annotation) were used to build phylogenetic trees with the Alakazam package. The *buildPhylipLineage()* function was executed, which is a wrapper around the dnapars binary, i.e. a maximum parsimony algorithm as implemented by the Phylip package [17]. Mutations with respect to germline were counted for all nodes using the *observedMutations()* function from the SHazaM package, which allowed counting in CDR and framework regions (FWRs) for both silent and non-silent mutations. For glycosylation prediction, Ig nucleotides were first translated to protein sequences. Reading frames were set by the start of the junctions/CDR3 in each receptor sequence. Subsequently, the fasta files of these protein sequences were analysed using the NetNGlyc 1.0 Server [18], without choosing (checking) all Asn residues. For each lineage, glycosylation sites, if any, were extracted and used to annotate nodes on the phylogenetic trees. Visualization of the trees was done using the *igraph* package [19].

Purification of serum anti-Ro60, anti-Ro52, anti-La and RF immunoglobulins

Serum anti-Ro60, anti-Ro52 and anti-La autoantibodies were purified using agarose gel immunodiffusion method as described previously [20]. Briefly, serum precipitin reactions between native Ro60, recombinant Ro52 or native La (Arotec Diagnostics, New Zealand) and serum from SjS patients were performed in 1% agarose gel (SeaKem, Lonza, MD, USA) at 37°C for 48 h. Precipitin lines were excised after extensive wash with distilled water, solubilised by boiling at 95°C for 5 min in the presence of 1% sodium dodecyl sulphate (SDS), and fractionated by SDS-polyacrylamide gel electrophoresis (PAGE) (Criterion stain-free TGX gels; Bio-Rad, Hercules, CA, USA). Serum RFs were purified from patient serum by a heat-aggregated IgG precipitation method, and separated on a reduced SDS-PAGE, as described previously [21].

Mass spectrometry (MS) sequencing and protein sequence data analysis

The immunoglobulin gel bands were excised and digested with trypsin (ThermoFisher Scientific, MA, USA), chymotrypsin and elastase (Promega, Madison, Wisconsin, USA), respectively. The digested peptides were subject to a Q Exactive HF-X (ThermoFisher Scientific) coupled to an Ultimate 3000 UHPLC (Dionex, Sunnyvale, California, USA). To identify matched clonotypic CDR3 peptides, peptide sequences derived from each individual purified autoantibodies were searched against the paired mRNA/DNA reference databases generated from the same patient by PEAKS Studio v8.5 (Bioinformatics Solutions). Parameters for database searches, data refinement and matched CDR3 peptide assignments are as follows: an average local confidence score threshold of ≥ 75% and manually inspected to ensure correct assignments; an false discovery rate (FDR) threshold of 0.5%; a maximum of two missed cleavages; precursor tolerance of <15 parts per million; product ion tolerance of 0.02 Da; precursor charge state of +2 to +4; fixed modification carbamidomethylation; variable modifications oxidation and deamidation; a maximum of 3 modifications allowed; non-specific cleavage at one end. Purification of antigen-specific autoantibodies from individual sera was carried out on at least two independent occasions, and the purified immunoglobulins digested by trypsin, chymotrypsin and elastase from each purification were subjected to mass spectrometry as two technical replicates, respectively.

Statistical analysis

All analyzed data were not normally distributed. Accordingly, data were reported as median and interquartile range (IQR) if not normal. Kruskal-Wallis, with post-hoc Bonferroni correction for multiple comparisons, Wilcoxon signed rank, Mann Whitney U test were used for comparisons where appropriate. P-values < 0.05 were considered statistically significant. Prism 7 software (Graph Pad, San Diego, CA, USA) was used to perform the statistical tests.

Supplementary methods reference list

1. [dataset] Van Schaik BD, Balzaretti G, Broeren M, de Vries N, Thurlings RM. Data from: Autoreactive B cell repertoire in blood and tissues of patients with Sjogren's syndrome. NCBI, december 13, 2021. https://www.ncbi.nlm.nih.gov/bioproject/PRJNA788352
2. Zhang J, Kobert K, Flouri T, et al. PEAR: a fast and accurate Illumina Paired-End reAd mergeR. *Bioinformatics (Oxford, England)* 2014;30(5):614-20. doi: 10.1093/bioinformatics/btt593
3. Giudicelli V, Chaume D, Lefranc MP. IMGT/GENE-DB: a comprehensive database for human and mouse immunoglobulin and T cell receptor genes. *Nucleic Acids Res* 2005;33(Database issue):D256-61. doi: 33/suppl_1/D256 [pii] gki010 [pii] 10.1093/nar/gki010 [doi] [published Online First: 2004/12/21]

4. Li H, Durbin R. Fast and accurate long-read alignment with Burrows-Wheeler transform. *Bioinformatics* 2010;26(5):589-95. doi: btp698 [pii] 10.1093/bioinformatics/btp698 [doi] [published Online First: 2010/01/19]

5. Li H, Handsaker B, Wysoker A, et al. The Sequence Alignment/Map format and SAMtools. *Bioinformatics* 2009;25(16):2078-9. doi: btp352 [pii] 10.1093/bioinformatics/btp352 [doi] [published Online First: 2009/06/10]

6. Koboldt DC, Chen K, Wylie T, et al. VarScan: variant detection in massively parallel sequencing of individual and pooled samples. *Bioinformatics* 2009;25(17):2283-5. doi: btp373 [pii] 10.1093/bioinformatics/btp373 [doi] [published Online First: 2009/06/23]

7. Klarenbeek PL, Tak PP, van Schaik BDC, et al. Human T-cell memory consists mainly of unexpanded clones. *Immunology Letters* 2010;133(1):42-48. doi: https://doi.org/10.1016/j.imlet.2010.06.011
8. Scheijen B, Meijers RWJ, Rijntjes J, et al. Next-generation sequencing of immunoglobulin gene rearrangements for clonality assessment: a technical feasibility study by EuroClonality-NGS. *Leukemia* 2019;33(9):2227-40. doi: 10.1038/s41375-019-0508-7

9. Bystry V, Reigl T, Krejci A, et al. ARResT/Interrogate: an interactive immunoprofiler for IG/TR NGS data. *Bioinformatics* 2017;33(3):435-37. doi: 10.1093/bioinformatics/btw634
10. Knecht H, Reigl T, Kotrová M, et al. Quality control and quantification in IG/TR next-generation sequencing marker identification: protocols and bioinformatic functionalities by EuroClonality-NGS. *Leukemia* 2019;33(9):2254-65. doi: 10.1038/s41375-019-0499-4
11. [dataset] 55. Broeren M, Pruijn G, Thurlings RM. Data from: Immune profiling of salivary gland and lymph node in Sjogren's Syndrome. NCBI, june 29, 2021. https://dataview.ncbi.nlm.nih.gov/object/PRJNA742201

11. [dataset] Broeren M, Pruijn G, Thurlings RM. Data from: Immune profiling of salivary gland and lymph node in Sjogren's Syndrome. NCBI, june 29, 2021. https://dataview.ncbi.nlm.nih.gov/object/PRJNA742201
12. Giudicelli V, Chaume D, Jabado-Michaloud J, et al. Immunogenetics Sequence Annotation: the Strategy of IMGT based on IMGT-ONTOLOGY. *Stud Health Technol Inform* 2005;116:3-8. [published Online First: 2005/09/15]

13. Gupta NT, Vander Heiden JA, Uduman M, et al. Change-O: a toolkit for analyzing large-scale B cell immunoglobulin repertoire sequencing data. *Bioinformatics (Oxford, England)* 2015;31(20):3356-58. doi: 10.1093/bioinformatics/btv359
14. Stern JNH, Yaari G, Vander Heiden JA, et al. B cells populating the multiple sclerosis brain mature in the draining cervical lymph nodes. *Science Translational Medicine* 2014;6(248):248ra107-248ra107. doi: 10.1126/scitranslmed.3008879
15. Yaari G, Uduman M, Kleinstein SH. Quantifying selection in high-throughput Immunoglobulin sequencing data sets. *Nucleic Acids Research* 2012;40(17):e134-e34. doi: 10.1093/nar/gks457
16. Yaari G, Uduman M, Kleinstein SH. Quantifying selection in high-throughput Immunoglobulin sequencing data sets. *Nucleic Acids Res* 2012;40(17):e134. doi: gks457 [pii] 10.1093/nar/gks457 [doi] [published Online First: 2012/05/30]

17. Bernard RB. PHYLIP: Phylogeny Inference Package. Version 3.2. Joel Felsenstein. *The Quarterly Review of Biology* 1989;64(4):539-41. doi: 10.1086/416571
18. Gupta R, Jung E, Brunak S. Prediction of N-glycosylation sites in human proteins. 2004;46:203-06.
19. Csárdi G, Nepusz T. The igraph software package for complex network research. *Conference Proceedings* 2006
20. Wang JJ, Colella AD, Beroukas D, et al. Precipitating anti-dsDNA peptide repertoires in lupus. *Clinical & Experimental Immunology* 2018;194(3):273-82. doi: 10.1111/cei.13197
21. Wang JJ, Reed JH, Colella AD, et al. Molecular Profiling and Clonal Tracking of Secreted Rheumatoid Factors in Primary Sjögren's Syndrome. *Arthritis & Rheumatology* 2018;70(10):1617-25. doi: 10.1002/art.40539

Supplementary Figure 1: Ig analysis strategy.

BM = bone marrow; Ig = immunoglobulin; Ig-DNA-seq = heavy and light chain immunoglobulin DNA analysis using next generation sequencing; Ig-RNAseq = heavy chain immunoglobulin RNA analysis using next generation sequencing; Ig-sc-seq = single-cell heavy and light chain immunoglobulin RNA sequencing; MALT = Mucosa Associated Lymphoid Tissue lymphoma; MS-seq = mass-spectrometry guided autoantibody analysis; post-RTX = tissue at clinical relapse after rituximab treatment; SG = salivary gland.

Supplementary Figure 2: Longitudinal samples and analyses in patients B005 (A) and B007 (B).

For patient B005 the salivary gland aspirate before rituximab treatment was acquired from an enlarged parotid gland during diagnostic evaluation. The salivary gland biopsy after rituximab was acquired from a sublingual gland for study purposes. AutoAb MS-seq = mass-spectrometry guided autoantibody analysis; Ig = immunoglobulin; Ig-DNA-seq = heavy and light chain immunoglobulin DNA analysis using next generation sequencing; Ig-RNAseq = heavy chain immunoglobulin RNA analysis using next generation sequencing; Ig-sc-seq = single-cell heavy and light chain immunoglobulin RNA sequencing.

Supplementary figure 3: histological analyses salivary gland and MALT lymphoma tissue biopsies.

Histological analyses were performed to analyse B and plasma cell infiltration in biopsies of affected tissues. As an example the salivary gland biopsy of B008 shows (A) a mixed infiltrate of leucocytes with haematoxylin and eosin stain; (B) periductular B-cell aggregates with CD20 staining; (C) kappa and (D) lambda positive intralobular plasma cells with kappa and lambda staining; breast needle and lung needle biopsies of two concomitant MALT

lymphomas of patient B013: (E) haematoxylin and eosin stain of a subcutaneous tissue needle biopsy shows remnants of germinal centres (arrows) surrounded by broad areas of small B-cells and sheets of plasma cells; (F) haematoxylin and eosin stain of a lung needle biopsy shows many extended B-cell follicles and septal infiltration by plasma cells; (G, K) kappa and lambda staining does not show light chain restriction.

Supplementary Figure 4: highly expressed B cell receptor sequences in tissues compared to blood and autoreactive clones in individual tissues.

Highly expressed B cell receptor sequences (HESs) were analysed in tissues compared blood samples. (A) the percentage of B cell clones with somatic hypermutations (SHM load > 0) in whole blood (WB) samples, circulating switched memory (SM), unswitched memory (UM), double negative (DN) B cells, affected salivary gland (SG) and MALT lymphoma (MALT) tissues; (B) the percentage of shared clones and (C) shared HESs between tissue specimens and whole blood and blood B cell subsets (PB = plasma blasts and BM bone marrow); (D) number of Ig-RNA clones in tissues that mapped to sequences of serum anti-Ro52, anti-La, anti-Ro60 and RF autoantibodies per patient. *=p<0.05.**=p<0.01, ***=p<0.001.

Supplementary Figure 5: Analytical controls.

B cell subsets were sorted from 6 SjS patients and 4 age-matched HCs. The sorted cells were naïve B cells (CD19+CD38-IGD+CD27-), unswitched memory (UM) cells (CD19+CD38-IGD+CD27+), double-negative (DN) B cells (CD19+CD38-IGD-CD27-), switched memory (SM) B cells (CD19+CD38-IGD-CD27+) and plasmablasts (PB)(CD20-CD38+CD138+). PBs were only obtained from SjS patients. (A) Total numbers of FACS-sorted cells. (B) Ig-RNAseq analysis: percentages of Ig HESs that were unique for a specific memory B cell subset. (C) Ig-RNAseq analysis: SHM load for the repertoire of B cell subsets of HCs. ***=p<0.001.

Supplementary Figure 6: IGHV usage in B cell subsets of healthy controls and SjS patients

A comparison was made in the top 10 IGHV gene segments used by naïve, marginal zone (MZ), double negative (DN), switched memory (memory) and plasmablasts (PB) in young healthy controls (HC), elderly HCs and SjS patients. No significant differences were found.

Supplementary Figure 7: IGHV usage in healthy controls versus SjS patients for memory B cell subsets

A comparison was made in the top 10 IGHV gene segments used by SjS patients versus young healthy controls (HC) and elderly HCs for naïve B cells, marginal zone (MZ), double negative (DN) and switched memory (memory) B cells. No significant differences were found.

Supplementary Figure 8: IGHV usage in expanded clones in memory B cell subsets of healthy controls versus SjS patients

A comparison was made in all IGHV gene segments used by SjS patients versus young healthy controls (HC) and elderly HCs for expanded clones (HECs) in marginal zone (MZ), double negative (DN) and switched memory (memory) B cells. No preferred IGHV usage was found.

Supplementary Figure 9: Presence of stereotypic RF in memory B cell subsets of healthy controls versus SjS patients

An analysis was made in the presence of known stereotypic V-J subfamily combinations used by RF in SjS related MALT Lymphoma (VH1.69-JH4, VH3.7-JH3, VH4.59-JH2). Vh1.69-JH4 was used by RF, including an RF expressing MALT lymphoma in of 2 of the 7 SjS patients in this analysis. A comparison was made for the presence of stereotypic RF in naïve, marginal zone (MZ), double negative (DN) versus switched memory (memory) B cells in SjS patients versus young healthy controls (HC) and elderly HCs. No enriched presence of stereotypic RF was found, except for an increase presence of Vh3.7-JH3 in SM B cells of young healthy controls * = P < 0.05.

Supplementary Figure 10: Presence of stereotypic RF in B cell subsets of healthy controls versus SjS patients

An analysis was made for the presence of known stereotypic V-J subfamily combinations used by RF in SjS related MALT lymphoma (VH1.69-JH4, VH3.7-JH3, VH4.59-JH2). Vh1.69-JH4 was used by RF, including an RF expressing MALT lymphoma in of 2 of the 7 SjS patients in this analysis. A comparison was made in for stereotypic RF enrichment in SjS patients versus young healthy controls (HC) and elderly HCs for expanded clones (HECs) in marginal zone (MZ), double negative (DN) and switched

memory (memory) B cells. No enrichment of stereotypic RF was found, except for an increased presence of Vh3.7-JH3 in DN B cells of SjS patients versus young and elderly healthy controls * = P < 0.05.

Supplementary Figure 11: Cluster analysis of cell populations from MALT lymphoma containing tissues. (A) Single-cell gene expression analysis of pooled duplicate single-cell RNA sequencing (scRNAseq) data of the SG of patient B005 (left) and the LN of patient B007 (right) at clinical relapse after RTX. The t-SNE plot shows clusters of cells that could be associated with specific cell types based on gene expression. A cluster of proliferating cells is indicated by a circle (left) and as GC B cells (right). **(B)** Dispersion of the most common RF clone over the different clusters in both tissues. The cells associated with the most common RF clone are depicted with bold black dots (left CATSST, right CAREMD).
